# Amot and Yap1 regulate neuronal dendritic tree complexity and locomotor coordination in mice

**DOI:** 10.1371/journal.pbio.3000253

**Published:** 2019-05-01

**Authors:** Katarzyna O. Rojek, Joanna Krzemień, Hubert Doleżyczek, Paweł M. Boguszewski, Leszek Kaczmarek, Witold Konopka, Marcin Rylski, Jacek Jaworski, Lars Holmgren, Tomasz J. Prószyński

**Affiliations:** 1 Nencki Institute of Experimental Biology, Polish Academy of Sciences, Warsaw, Poland; 2 Centre of Postgraduate Medical Education, Warsaw, Poland; 3 Institute of Psychiatry and Neurology, Warsaw, Poland; 4 International Institute of Molecular and Cell Biology, Warsaw, Poland; 5 Karolinska Institutet, Stockholm, Sweden; University of Basel, SWITZERLAND

## Abstract

The angiomotin (Amot)–Yes-associated protein 1 (Yap1) complex plays a major role in regulating the inhibition of cell contact, cellular polarity, and cell growth in many cell types. However, the function of Amot and the Hippo pathway transcription coactivator Yap1 in the central nervous system remains unclear. We found that Amot is a critical mediator of dendritic morphogenesis in cultured hippocampal cells and Purkinje cells in the brain. Amot function in developing neurons depends on interactions with Yap1, which is also indispensable for dendrite growth and arborization in vitro. The conditional deletion of Amot and Yap1 in neurons led to a decrease in the complexity of Purkinje cell dendritic trees, abnormal cerebellar morphology, and impairments in motor coordination. Our results indicate that the function of Amot and Yap1 in dendrite growth does not rely on interactions with TEA domain (TEAD) transcription factors or the expression of Hippo pathway–dependent genes. Instead, Amot and Yap1 regulate dendrite development by affecting the phosphorylation of S6 kinase and its target S6 ribosomal protein.

## Abstract 2

Amot and Yap1 collaborate to organize neuronal dendritic trees in a process that involves regulation of the S6 kinase–dependent phosphorylation of S6 ribosomal protein; conditional Amot and Yap1 knockout mice exhibit abnormalities in cerebellar morphology and impairments in locomotor coordination.

## Introduction

Neurons are highly polarized cells with specialized axonal and somatodendritic compartments. The architecture of dendritic arbors where information from other neurons is received is crucial for the integration and processing of signals [1,2]. Although characteristic dendritic patterns are driven by both intrinsic mechanisms and external signals [3–5], the molecular processes that underlie the formation of dendritic trees have been studied mostly in cultured hippocampal neurons under conditions in which external signals are limited. Abnormalities in dendritic arborization are associated with numerous neurological disorders, such as schizophrenia, epilepsy, Alzheimer disease, and autism spectrum disorder [6–10]. Thus, a better understanding of the molecular processes that regulate dendritic tree patterning could facilitate the development of new treatment modalities for various neurological and psychiatric conditions.

Angiomotin (Amot) protein and the closely related angiomotin-like 1 (Amotl1) and angiomotin-like 2 (Amotl2) proteins constitute a family of scaffold proteins called angiomotins or motins [11,12]. Amot is the most characterized member of the angiomotin family. This protein was shown to regulate many physiological and pathological processes, including angiogenesis, cell polarity, cell migration, and cancer cell progression [13–19]. However, one of the most characterized functions of Amot and other motins is regulation of the Hippo signaling pathway.

The Hippo signaling pathway controls cellular mechanotransduction, cell growth, and cell differentiation [20–23]. A core component of this pathway is the cascade of kinases, mammalian sterile 20-like kinase (Mst) and large tumor suppressor kinase (Lats), that regulate phosphorylation of the transcription coactivators Yes-associated protein 1 (Yap1) and Taz, two major downstream effectors of the Hippo pathway [20,21,24–27]. Phosphorylation deactivates Yap1 by inhibiting its translocation to the nucleus [20,21], where it interacts with TEA domain (TEAD) transcription factors to induce the expression of Hippo pathway–dependent genes [20,28,29]. Amot has been shown to directly and strongly interact with Yap1 [30]. The function of Amot in the regulation of Yap1 appears to be both tissue- and cell type–specific [16,30–32]. For example, in MDCK epithelial cells and human embryonic kidney 293 (HEK-293) cells, Amot inhibits Yap1-dependent transcription. In contrast, in hepatocarcinoma and breast cancer cells, Amot induces the transcription of TEAD target genes [16,30–32]. Both Amot and Yap1 have been shown to regulate the contact-mediated inhibition of cell proliferation and control organ size and growth [17,21,22,30].

The function of Amot protein in neurons is poorly understood. A recent study reported that Amot is localized to dendritic spines in cultured hippocampal neurons, where it controls integrity of the postsynaptic density by regulating multi-PDZ domain protein 1 (MUPP1) and postsynaptic density-95 (PSD-95) [33]. Schanzenbächer and colleagues [34] reported that Amot is associated with neuronal homeostatic scaling and autism spectrum disorder. In the present study, we report that Amot is enriched at synapses of mature neurons. At earlier developmental stages, however, Amot is localized to dendritic processes and is required for the proper growth and development of dendritic arbors both in vitro and in vivo. We found that Amot in neurons interacts with Yap1. Similar to Amot, Yap1 localizes to neurites in young neurons and translocates to synapses in mature cells. We found that Yap1 is also critical for dendritic tree growth and arborization. Our results suggest that the mechanism by which Amot and Yap1 regulate dendrite development does not depend on the Yap1–TEAD interaction or regulation of the expression of Hippo pathway–controlled genes. Instead, we demonstrated that Amot and Yap1 maintain the proper formation of dendritic arbors by regulating the phosphorylation of S6 ribosomal protein by S6 kinase (S6K).

## Results

### Amot expression and localization in neurons

**Fig 1:**
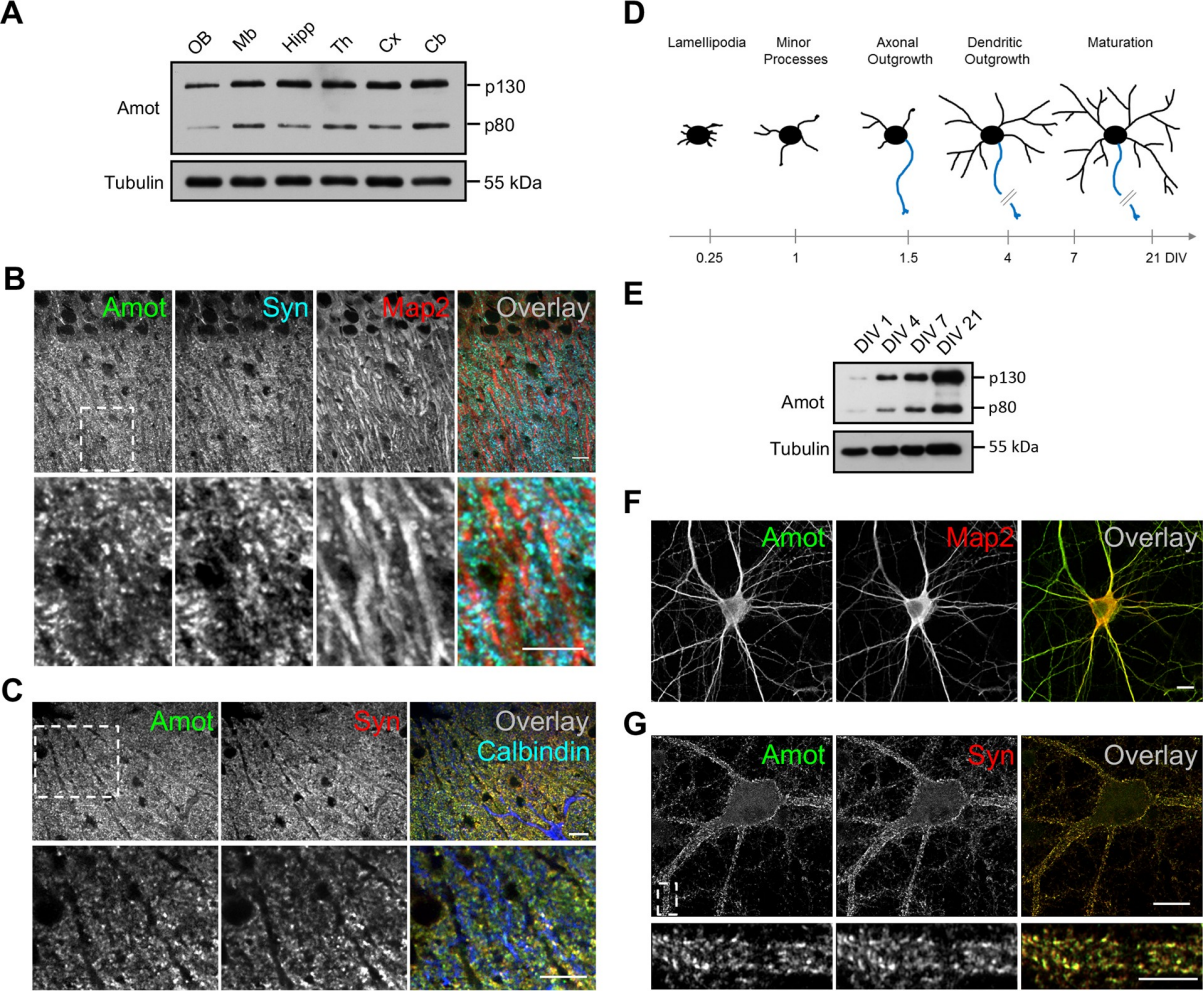
Expression of angiomotin proteins in neurons. (A) Western blot analysis of Amot expression in different regions of the mouse brain. “OB” = olfactory bulbs; “Mb” = midbrain; “Hipp” = hippocampus; “Th” = thalamus; “Cx” = cortex; “Cb” = cerebellum. (B, C) Amot localization in neurons in cryostat sections of the P30 mouse brain. (B) CA1 region of the hippocampus stained for Amot (green), Syn (blue), and Map2 (red). (C) Purkinje cell dendritic trees in the cerebellum stained for Amot (green), Syn (red), and calbindin (blue). (D) Model of hippocampal neuron development in vitro. Development of the somatodendritic compartment (black) and axon (blue) is correlated with DIVs on the axis at the bottom. (E) Amot expression increases at later stages of neuronal development. Neuronal extracts that were collected on the indicated DIVs were analyzed by western blot. (F, G) Localization of Amot protein in cultured neurons. (F) Rat hippocampal neurons (DIV7) were immunolabeled for Amot (green) and Map2 (red). (G) Amot localization in DIV21 cultured rat hippocampal neurons, showing Amot (green) and Syn (red). The lower panels in B, C, and G show higher magnifications of the boxed areas in the upper panels. Scale bar = 10 μm in B, C, F, and G (upper panel) and 5 μm in G (lower panel). Amot, angiomotin; DIV, day in vitro; Map2, microtubule-associated protein 2; P, postnatal day; Syn, synaptophysin.

Amot has been previously shown to be ubiquitously expressed in various rat brain structures and localized to synapses in cultured mature neurons [33]. Our western blot analysis of brain homogenates revealed high levels of Amot expression in different regions of the mouse brain (Fig 1A). The full-length isoform of Amot (p130) was predominantly expressed in the brain, but the shorter isoform (p80) that lacked the N′-terminal domain was also produced in lower amounts. To study Amot localization in the brain, we immunohistochemically analyzed cryostat brain sections. These experiments revealed that Amot is concentrated at synapses in the CA1 region of the hippocampus (Fig 1B) and Purkinje cells in the cerebellum (Fig 1C and S1B Fig), where its immunoreactivity overlaps with anti-synaptophysin staining.

We next studied the detailed expression and localization of Amot in hippocampal neurons that were cultured in vitro. The western blot analysis revealed that Amot expression gradually increased during neuronal maturation (Fig 1D and 1E) and was particularly high at the time of intensive dendritogenesis on days 4–7 in vitro (DIV4–7). The increase in expression was similar for both isoforms of Amot (p130 and p80). Our immunocytochemical analysis of cultured rat hippocampal neurons on DIV7 showed that Amot at this developmental stage was mostly concentrated in dendrites and axons (Fig 1F). Similar localization to dendrites was observed in DIV7 hippocampal neurons that overexpressed the green fluorescent protein (GFP)-tagged version of Amot (S1 Fig), although, as reported previously, Amot–GFP fusion protein showed a more scattered distribution [35]. At later stages of neuronal development (DIV21), Amot relocalized to synaptic compartments, where it colocalized with synaptophysin puncta, revealed by Airyscan microscopy (Fig 1G).

### Amot regulates dendritic tree development in cultured hippocampal neurons

**Fig 2:**
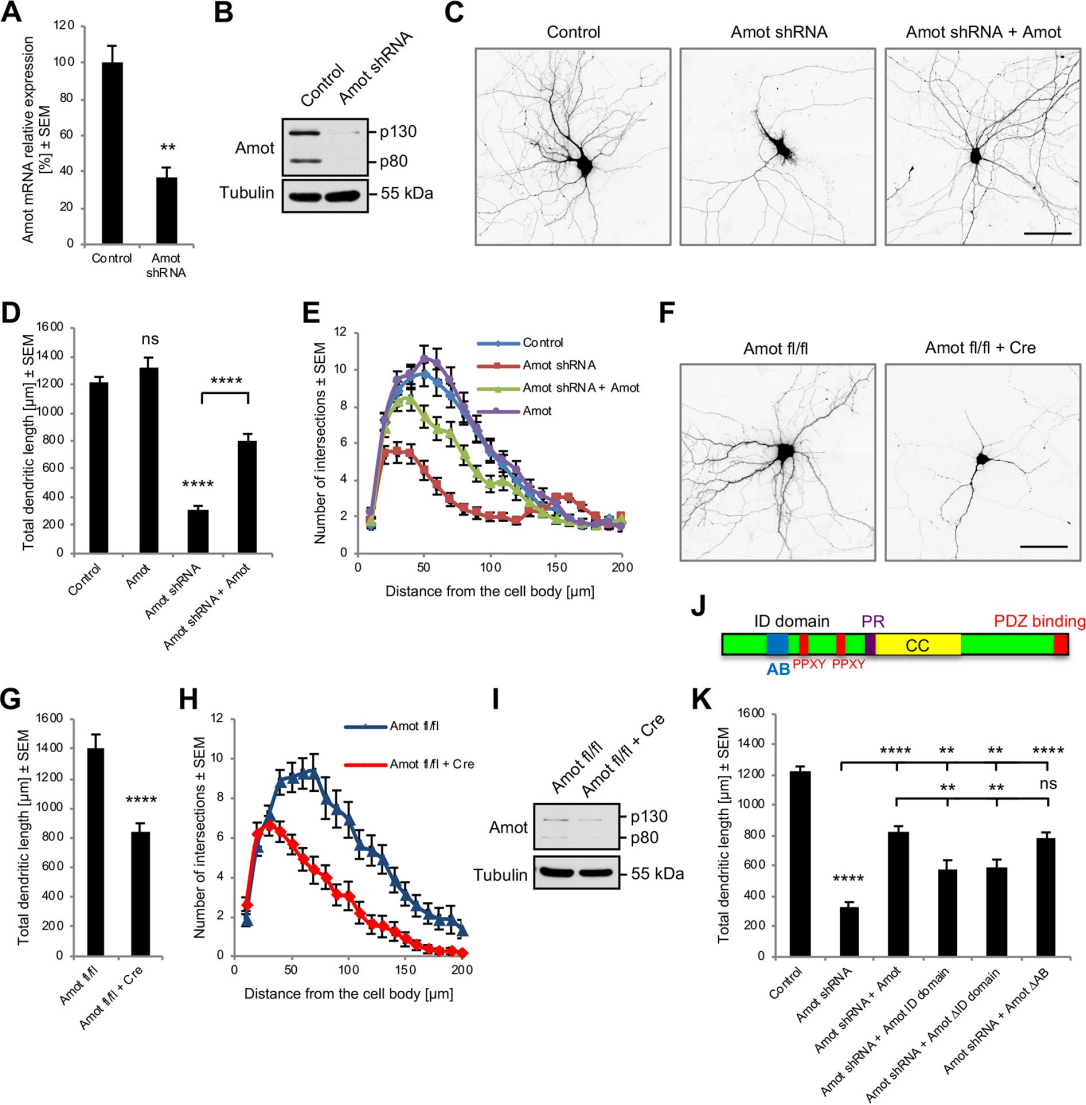
Amot depletion in cultured hippocampal neurons affects dendritic tree organization. (A, B) Validation of Amot-knockdown efficiency in neurons by qRT-PCR (*n* = 3/group; *p* = 0.0041) (A) and western blot (B). (C) Rat hippocampal neurons were transfected on DIV7 with an empty pLKO plasmid (control), a plasmid with Amot shRNA, and a plasmid with Amot shRNA and a plasmid that expressed mouse Amot–GFP (Amot shRNA + Amot; rescue experiment). (D, E) Quantification of TDL (D) and Sholl analysis (E) of hippocampal neurons that were transfected with plasmids as in (C) or transfected with Amot–GFP plasmid. Control: *n* = 60; Amot: *n* = 38; Amot shRNA: *n* = 60; Amot shRNA + Amot: *n* = 58. To Control; *p* = 0.2678, *p* < 0.0001; to Amot shRNA; *p* < 0.0001. (F) Reduction of dendritic tree complexity in hippocampal neurons from *Amot fl/fl* mice that were transfected with a Cre-expressing plasmid. (G, H) Quantification of TDL (G) and Sholl analysis (H) of dendritic trees of neurons from (F). Control vector: *n* = 33; Cre plasmid: *n* = 40. *p* < 0.0001. (I) Cre expression in *Amot fl/fl* hippocampal neurons led to the down-regulation of Amot expression, determined by western blot. (J) Domain architecture of Amot protein. The Amot ID domain contains LPXY and PPXY motifs (red) that bind Yap1 and the AB (blue). (K) Quantification of TDL of Amot-knockdown rat hippocampal neurons that were transfected with the indicated Amot constructs. Control cells were transfected with an empty pLKO vector. Control: *n* = 107; Amot shRNA: *n* = 80; Amot shRNA + Amot: *n* = 100; Amot shRNA + Amot ID domain: *n* = 37; Amot shRNA + Amot ΔID domain: *n* = 45; Amot shRNA + Amot ΔAB: *n* = 59. To control; *p* < 0.0001; to Amot shRNA; *p* < 0.0001, *p* = 0.0064, *p* = 0.0015, *p* < 0.0001; to Amot shRNA + Amot; *p* = 0.0047, *p* = 0.0033, *p* = 0.09695. Images were obtained from at least three independent cultures. Statistical significance was analyzed using two-tailed and unpaired *t* tests (A, G), one-way analysis of variance followed by Tukey’s post hoc test (D, K), and two-way analysis of variance followed by Bonferroni’s test (E, H). Numerical values that underlie the graphs are shown in S1 Data. See S1 Table for detailed statistics for E and H. **p < 0.01, ****p < 0.0001. Bars represent the mean ± SEM. Scale bars = 50 μm. AB, actin-binding motif; Amot, angiomotin; CC, coiled-coil domain; DIV, day in vitro; GFP, green fluorescent protein; ID, intrinsic disordered; ns, not significant; PR, proline-rich region; qRT-PCR, quantitative real-time polymerase chain reaction; SEM, standard error of the mean; shRNA, short-hairpin RNA; TDL, total dendrite length; Yap1, Yes-associated protein 1.

Amot’s localization within dendritic and axonal compartments during neuronal maturation and its higher expression at the time of neurite outgrowth suggest that it could regulate the development of neuronal processes. To investigate this possibility, we performed RNA interference (RNAi)-mediated silencing of Amot expression in DIV7 cultured hippocampal neurons. We cloned short-hairpin RNA (shRNA) plasmids and selected the one with the highest knockdown efficiency in neurons, which was confirmed by quantitative real-time polymerase chain reaction (qRT-PCR) and western blot (Fig 2A and 2B). To visualize the morphology of individual neurons, we cotransfected cells with the shRNA and a plasmid that expressed monomeric red fluorescent protein (mRFP) under the control of β-actin regulatory elements. As expected, neurons that were transfected with Amot shRNA plasmid had severe defects in dendritic tree arborization compared with control cells that were transfected with an empty vector (Fig 2C). These cells had an approximately 75% shorter total dendrite length (TDL; sum of all dendrite lengths) compared with control neurons (Fig 2D) and formed significantly less-complex dendritic arbors, verified by Sholl analysis (Fig 2E). Sholl analysis is used to indicate the number of dendrites that cross circles at various radial distances from the cell soma and describes dendritic arbor complexity and the area of a dendritic field [36]. The observed phenotypes of neuron morphology were specific to Amot rather than an off-target effect of shRNA overexpression because the phenotype could be reversed (i.e., rescued) by the ectopic expression of a mouse Amot construct that lacked a nucleotide sequence that was targeted by shRNA (Fig 2C, 2D and 2E). The overexpression of Amot alone had no significant effects on dendritic tree development (Fig 2D and 2E).

To independently confirm the involvement of Amot in dendritic tree organization, we examined the morphology of cultured hippocampal neurons that were obtained from conditional *Amot* knockout mice (*Amot fl/fl*; see description in the section “Characterization of *Amot fl/fl;Syn-Cre* mice”) that were cotransfected with a GFP-encoding plasmid to visualize their morphology and either a Cre recombinase–expressing plasmid or control plasmid without Cre (Fig 2F). Neurons that were transfected with the Cre plasmid (S2A Fig) had an approximately 60% shorter TDL and simplified dendritic tree morphology, revealed by Sholl analysis (Fig 2G and 2H). Amot-depleted neurons maintained polarized distribution of the dendritic marker microtubule-associated protein 2 (Map2) (S3A Fig) and axon initial segment protein ankyrin G (S3B Fig). The formation of axons in Cre-transfected neurons was also unaffected (S3C Fig). To confirm the reduction of Amot expression, we performed western blot analysis of extracts from *Amot fl/fl* cortical neurons that were nucleofected with a plasmid that encoded Cre recombinase or a control vector. We found that Cre expression in *Amot fl/fl* neurons reduced Amot protein levels (Fig 2I), although some residual expression was still detected, likely because of the roughly 50% transfection efficiency in the nucleofection experiments. Cre expression in cultured neurons that were obtained from wild-type mice did not exhibit a decrease in Amot expression and had no effect on dendritic topology (S2B, S2C and S2D Fig). Altogether, these results support the conclusion that Amot is required for proper dendritic arbor morphology in developing hippocampal neurons. The loss of Amot function in mature neurons also compromised dendritic organization, but the defect appeared to be less pronounced compared with developing cells (S2F Fig).

To identify the Amot protein region that is responsible for regulating dendritic topology, we rescued the Amot-knockdown phenotype by ectopically expressing truncated Amot constructs. The N-terminal intrinsic disordered (ID) domain of Amot (Fig 2J) is known to directly interact with F-actin through a 34-amino-acid sequence [35] and contains two PPXY motifs that bind the Hippo pathway transcription coactivator Yap1 [30,31]. The transfection of Amot-depleted neurons with constructs that encoded either the ID domain alone (Amot ID) or Amot with ID domain deletion (Amot ΔID) led to the partial restoration of dendritic tree arborization compared with the expression of full-length Amot (Fig 2K). This observation suggests that the Amot ID domain plays an important role in regulating dendritic processes, but other domains could also be involved. We next investigated whether the Amot-regulated organization of neuronal dendritic trees depends on the actin-binding site [35,37] that is centrally located within the ID domain (Fig 2J). The expression of Amot mutant protein with a deleted actin-binding region (Amot ΔAB) rescued the phenotype of Amot depletion to an extent that was comparable to the expression of full-length Amot protein (Fig 2K). All of the Amot constructs that were used in the phenotype rescue experiments were expressed in neurons at similar levels (S4A Fig). Some of the expressed proteins had a more punctate distribution, whereas others were more evenly distributed. However, all of the proteins were localized to dendritic processes (S4D Fig). Nevertheless, we cannot exclude the possibility that the slight differences in expression levels and alterations of localization of the Amot ΔID construct could contribute to the modest rescue phenotype that was observed. These results suggest that the interaction with actin does not play an important role in the Amot-dependent regulation of dendritic trees.

### Amot interaction with Yap1 is critical for dendrite morphogenesis

**Fig 3:**
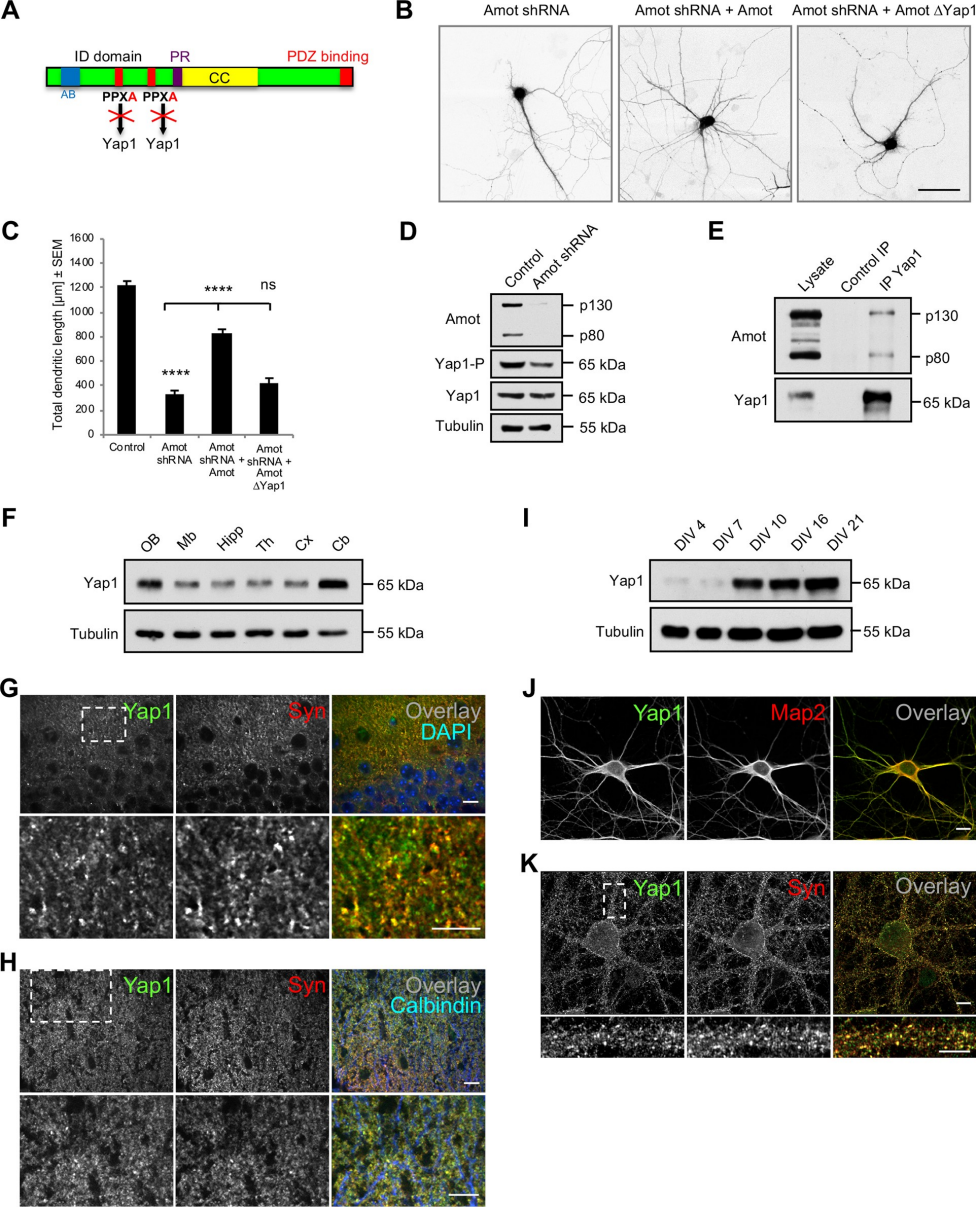
Amot interaction with Yap1 is required for proper growth of dendritic arbors. (A) Schematic illustration of Amot protein with mutated Yap1-binding sites where PPXY motifs were modified into PPXA. (B) Rat hippocampal neurons that were transfected on DIV7 with the indicated constructs. (C) Quantification of TDL of Amot-knockdown rat hippocampal neurons that were cotransfected with plasmids as in B. Control cells were transfected with an empty pLKO vector. Control: *n* = 107; Amot shRNA: *n* = 80; Amot shRNA + Amot: *n* = 100; Amot shRNA + Amot ΔYap1: *n* = 59. To control; *p* < 0.0001; to Amot shRNA *p* < 0.0001; *p* = 0.3853. Images were obtained from at least from three independent cultures. (D) Yap1 phosphorylation at serine-127 was visibly reduced in Amot-depleted neurons, whereas the total cellular level of Yap1 was unchanged. (E) Yap1 coprecipitated with Amot from mouse brain homogenates. Uncoated magnetic beads were used as the control. (F) Western blot analysis of Yap1 expression in different regions of the mouse brain. “OB” = olfactory bulbs; “Mb” = midbrain; “Hipp” = hippocampus; “Th” = thalamus; “Cx” = cortex; “Cb” = cerebellum. (G, H) Yap1 localization in the brain, showing cryostat sections of the P30 mouse hippocampus (G) and cerebellum (H) that were stained for Yap1 (green) and Syn (red). Overlay images additionally showed nuclei, visualized with DAPI (blue in G), and Purkinje cell dendritic processes, visualized with anti-calbindin antibody (blue in H). (I) Yap1 expression increased at later stages of neuronal development. Neuronal extracts that were collected on the indicated DIVs were analyzed by western blot. (J) Yap1 localized to dendritic processes in young cultured neurons. Rat hippocampal neurons (DIV8) were immunolabeled for Yap1 (green) and Map2 (red). (K) Yap1 localized to synapses in mature cultured neuronal cells. Rat hippocampal neurons (DIV21) were immunolabeled for Yap1 (green) and Syn (red). The lower panels in G, H, and K show higher magnifications of the boxed areas in the upper panels. Scale bars = 50 μm in B; 10 μm in G, H, J, and K (upper panel); and 5 μm in K (lower panel). Numerical values that underlie the graphs are shown in S1 Data. Statistical significance was analyzed using one-way analysis of variance followed by Tukey’s post hoc test. ****p < 0.0001. Bars represent the mean ± SEM. AB, actin-binding motif; CC, coiled-coil domain; DIV, day in vitro; ID, intrinsic disordered; IP, immunoprecipitation; Map2, microtubule-associated protein 2; ns, not significant; P, postnatal day; PR, proline-rich region; SEM, standard error of the mean; shRNA, short-hairpin RNA; Syn, synaptophysin; TDL, total dendrite length; Yap1, Yes-associated protein 1.

To study the importance of Yap1-binding motifs that are located in the Amot ID domain, we expressed in Amot-knockdown neurons a mutant of Amot that exhibited impairments in binding to Yap1 (Fig 3A). We confirmed that the Amot constructs that were used in the phenotype rescue experiments were expressed in neurons at similar levels (S4B Fig) and were localized to dendrites (S4D Fig). The expression of Amot that was defective in Yap1 binding (Amot ΔYap1) failed to rescue impairments in dendritic topology that were observed in Amot-depleted neurons (Fig 3B and 3C), suggesting that the interplay between Amot and Yap1 plays an important role in controlling dendritic tree formation. To further investigate this possibility, we analyzed whether Amot depletion in neurons is correlated with alterations of Yap1 expression or phosphorylation. As shown in Fig 3D, the total cellular level of Yap1 protein was similar in control and Amot-knockdown neurons. However, Yap1 phosphorylation at serine-127 (Ser127) substantially decreased in the absence of Amot (Fig 3D). Phosphorylation at Ser127 has been shown to modulate Yap1 function [21,38]. To verify the interaction between Amot and Yap1, we precipitated Yap1 from mouse brain extracts and found that Amot coprecipitated with Yap1 but not with control beads (Fig 3E), suggesting an interaction between these two proteins. This is consistent with previous studies that demonstrated a direct and strong interaction between Amot and Yap1 in other cell types [30–32].

Although Yap1 has been previously shown to be expressed in progenitor cells in the brain, where it regulates proliferation, migration, and differentiation under both physiological and pathological conditions, the function of Yap1 has not yet been characterized in vertebrate neurons [39–42]. Our western blot analysis revealed that Yap1 is widely expressed in different brain structures, with the highest levels in the cerebellum (Fig 3F). To study the localization of Yap1, we immunohistochemically analyzed cryostat sections of the mouse postnatal day (P)30 hippocampus (Fig 3G) and cerebellum (Fig 3H and S1C Fig). Yap1 immunoreactivity was observed predominantly at synaptic compartments, where it colocalized with synaptophysin puncta.

We next studied the expression and localization of Yap1 in hippocampal neurons that were cultured in vitro. The western blot analysis revealed that Yap1 expression increased after DIV7 (Fig 3I). The immunocytochemical analysis of cultured rat hippocampal neurons on DIV8 showed that Yap1 at this developmental stage was mostly concentrated in dendrites and axons (Fig 3J). At later stages of neuronal development (DIV21), Yap1 relocalized to synaptic compartments, where it colocalized with synaptophysin puncta, revealed by Airyscan microscopy (Fig 3K). Thus, Yap1 localization resembles Amot at both the early and late stages of neuronal development.

### Yap1 regulates dendritic tree organization

Yap1 localized to dendrites in developing hippocampal neurons, and the ectopic expression of Amot protein with mutated Yap1 binding sites failed to rescue the phenotypes that were observed in Amot-depleted cells. Therefore, we hypothesized that Amot may organize dendritic trees through Yap1.

**Fig 4:**
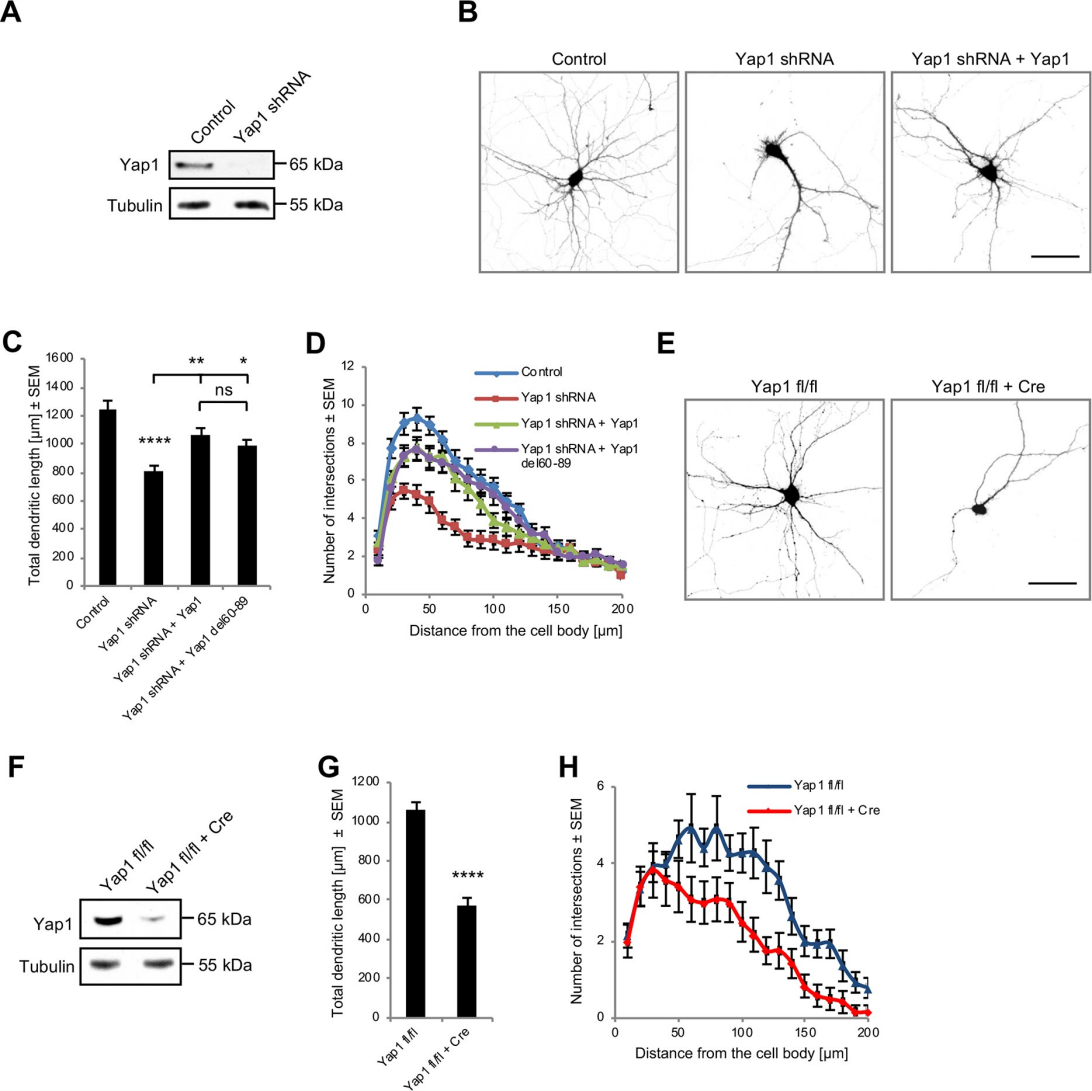
Yap1 regulates dendritic tree complexity. (A) Efficacy of Yap1 shRNA in neurons, analyzed by western blot. (B) Rat hippocampal neurons were transfected on DIV7 with the indicated constructs. An empty pLKO plasmid was used as the control. (C, D) Quantification of TDL (C) and Sholl analysis (D) of hippocampal neurons that were transfected with plasmids as in B or cotransfected with Yap1 shRNA and human Yap1-cDNA with a deleted TEAD-binding domain (Yap1 shRNA + Yap1 del60-89). Control: *n* = 40; Yap1 shRNA: *n* = 60; Yap1 shRNA + Yap1: *n* = 47; Yap1 shRNA + Yap1 del60-89: *n* = 51. To control; *p* < 0.0001; to Yap1 shRNA; *p* = 0.0011, *p* = 0.0264; to Yap1 shRNA + Yap1; *p* = 0.7244. (E) Reduction of dendritic tree complexity in hippocampal neurons from *Yap1 fl/fl* mice that were transfected with a Cre-expressing plasmid. (F) Cre expression in *Yap1 fl/fl* hippocampal neurons led to the down-regulation of Yap1 expression, analyzed by western blot. (G, H) Quantification of TDL (G) and Sholl analysis (H) of dendritic trees of hippocampal *Yap1 fl/fl* neurons that were transfected with either a control vector (*n* = 53) or a Cre-expressing plasmid (*n* = 53). *p* < 0.0001. Images were obtained from at least from three independent cultures. Statistical significance was analyzed using two-tailed unpaired *t* test (G), one-way analysis of variance followed by Tukey’s post hoc test (C), and two-way analysis of variance followed by Bonferroni’s post hoc test (D, H). Numerical values that underlie the graphs are shown in S1 Data. See S1 Table for detailed statistics for D and H. *p < 0.05, **p < 0.01, ****p < 0.0001. Bars represent the mean ± SEM. Scale bar = 50 μm. DIV, day in vitro; ns, not significant; SEM, standard error of the mean; shRNA, short-hairpin RNA; TDL, total dendrite length; TEAD, TEA domain; Yap1, Yes-associated protein 1.

To determine whether Yap1 plays a role in dendritic tree arbor development, we knocked down Yap1 expression in cultured rat hippocampal neurons by transfecting cells with shRNA, which has the highest knockdown efficiency in neurons (Fig 4A), and examined their morphology. Developing hippocampal neurons that were transfected with a plasmid that encoded shRNA that targeted rat Yap1 exhibited significant impairments in dendritic arbors, reflected by a 40% decrease in TDL and a shift in Sholl plots compared with control cells that were transfected with an empty vector (Fig 4B, 4C and 4D). The negative effects of Yap1 knockdown on dendritic arbors were almost fully reversed by the ectopic expression of human Yap1 cDNA, which lacks the nucleotide sequence targeted by used shRNA (Fig 4B, 4C and 4D). Similarly, the loss of Yap1 function in mature neurons compromised dendritic organization, but the defect appeared to be less pronounced compared with developing cells (S2F Fig).

One of the well-documented functions of Yap1 is the regulation of gene expression that is controlled by the Hippo signaling pathway. This depends on the interaction between Yap1 and TEAD transcription factors. Upon interactions with coactivators, TEAD transcription factors bind to TEA sequences in regulatory elements of many genes, stimulating their transcription [28,29]. To investigate whether the function of Yap1 in the organization of dendritic processes involves interactions with TEAD, we attempted to rescue phenotypes that were observed in Yap1 knockdown neurons by the ectopic expression of mutant Yap1 protein (Yap1 del60-89) that lacked the TEAD-binding domain [43]. Expression of the Yap1 del60-89 mutant also rescued the defects in dendritic tree patterning to an extent that was comparable to wild-type Yap1 (Fig 4C and 4D). In this experiment, both Yap1 constructs were expressed at similar levels (S4C Fig) and had similar localization in neurons (S4E Fig).

To independently confirm the role of Yap1 in dendritic tree growth, we examined the morphology of cultured hippocampal neurons that were obtained from *Yap1* conditional knockout mice (*Yap1 fl/fl*; see description in the section “Yap1 is required for cerebellar organization and Purkinje cell dendritogenesis”) and transfected with either a plasmid that expressed Cre recombinase or a control plasmid without Cre. We found that Cre expression in *Yap1 fl/fl* neurons decreased Yap1 expression, reduced TDL by approximately 40%, and simplified dendritic tree morphology, revealed by Sholl analysis (Fig 4E, 4F, 4G and 4H). Yap1-depleted neurons, however, maintained polarized distribution of the dendritic marker Map2 (S5A Fig) and axon initial segment protein ankyrin G (S5B Fig). The formation of axons in Cre-transfected neurons was also unaffected (S5C Fig). In control neurons that were obtained from wild-type mice, Cre expression did not affect Yap1 protein levels or dendritic tree organization (S2C, S2D and S2E Fig).

Altogether, our results suggest that Yap1 is expressed in neurons, where it regulates dendritic tree arborization in a way that appears to be independent of interactions with TEAD transcription factors.

### Characterization of *Amot fl/fl;Syn-Cre* mice

**Fig 5:**
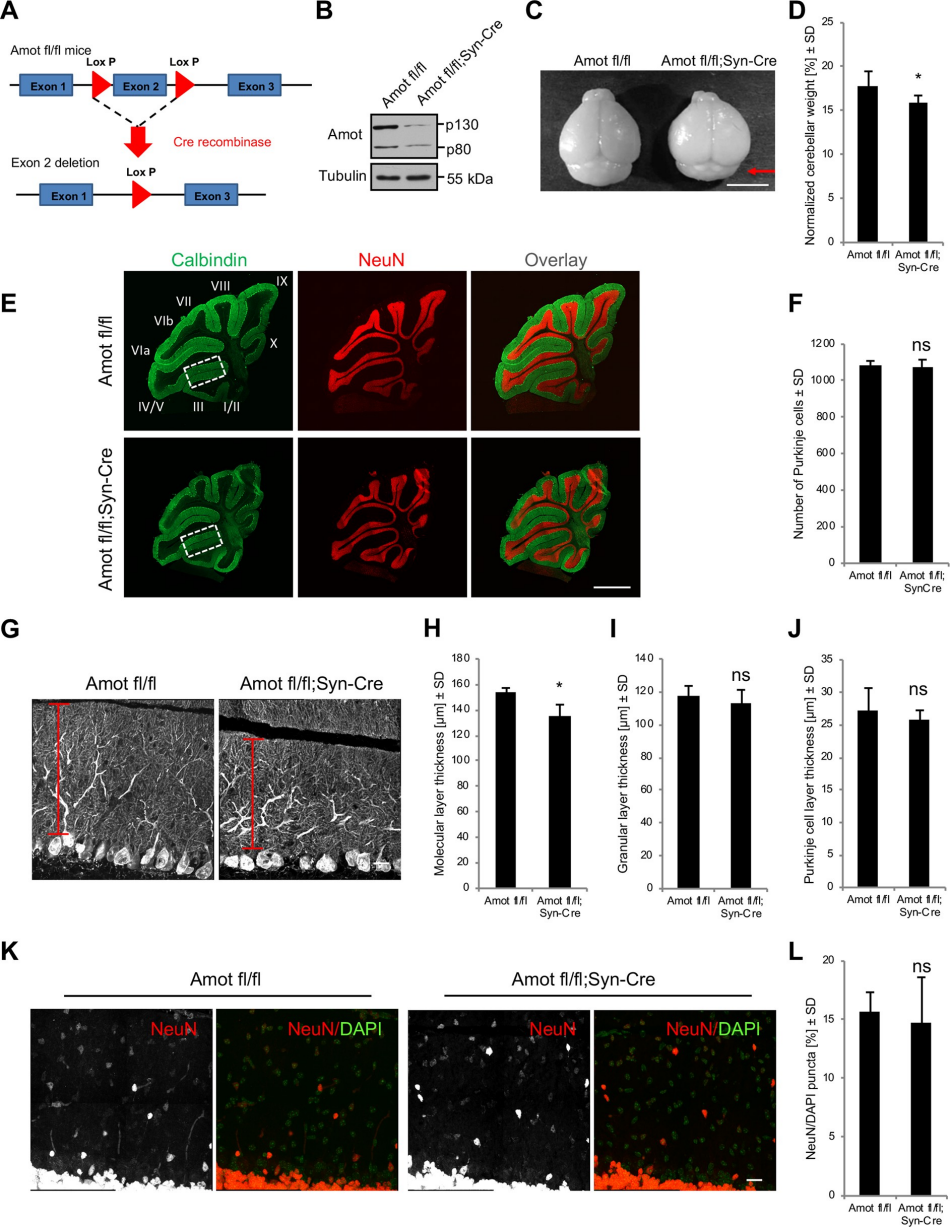
Neuronal deletion of Amot affects cerebellar morphology. (A) Strategy for the generation of *Amot* conditional knockout mice, showing the *Amot flox* allele (upper diagram) and recombined allele after Cre-mediated excision (lower diagram). (B) Lower Amot expression in the cerebellum of *Amot fl/fl;Syn-Cre* P30 mice, analyzed by western blot. (C) *Amot fl/fl;Syn-Cre* mice exhibited a smaller cerebellum compared with control *Amot fl/fl* littermates. (D) Quantitative analysis of cerebellar weight in *Amot fl/fl* (*n* = 9) and sex-matched *Amot fl/fl;Syn-Cre* (*n* = 6) mice on P30. *p* = 0.0411. The measurements were normalized to the whole-brain weight. (E) Sagittal cerebellar sections of *Amot fl/fl* and *Amot fl/fl;Syn-Cre* P30 mice that were immunolabeled for calbindin (green) to visualize Purkinje cells and the molecular layer and NeuN (red) to visualize the granule cell layer. Numbers indicate individual lobes. (F) Quantification of Purkinje cell number in sagittal sections of *Amot fl/fl* mice (*n* = 3 mice and at least three sections per mouse) and *Amot fl/fl;Syn-Cre* mice (*n* = 4 mice and at least three sections per mouse). *p* = 0.5405. (G) Decrease in molecular layer thickness in *Amot fl/fl;Syn-Cre* mice, visualized by anti-calbindin antibody; lobe III down the preculminate fissure. (H-J) Thickness of the molecular (H), granular (I), and Purkinje (J) cell layers in the cerebellum of *Amot fl/fl* mice (*n* = 3 mice and at least three sections per mouse) and *Amot fl/fl;Syn-Cre* mice (*n* = 5 mice and at least three sections per mouse) on P30 measured in lobe III down the preculminate fissure. *p* = 0.0135, *p* = 0.4169, and *p* = 0.4615. (K, L) Granule cell distribution (K) and quantification (L) in the molecular layer of the cerebellum in P30 *Amot fl/fl* mice (*n* = 3 mice and at least three sections per mouse) and *Amot fl/fl;Syn-Cre* mice (*n* = 5 mice and at least three sections per mouse), shown as a percentage of NeuN-positive cells per 20,000 μm^2^. *p* = 0.7333. Numerical values that underlie the graphs are shown in S1 Data. Statistical significance was analyzed using two-tailed unpaired *t* test. *p < 0.05. Bars represent the mean ± SD. Scale bars = 5 mm in C, 1,000 μm in E and 20 μm in G and K. Amot, angiomotin; ns, not significant; P, postnatal day; SD, standard deviation.

To investigate the role of Amot in the organization of neuronal cells in vivo, *Amot fl/fl* mice, in which exon 2 of *Amot* was flanked by LoxP sites (Fig 5A), were crossed with transgenic mice that expressed Cre recombinase under *Synapsin 1* regulatory elements (*Syn-Cre* mice), leading to Cre expression that was restricted to neuronal cells. To confirm the activity and specificity of recombination in *Syn-Cre* mice, we crossed them with a *STOP-tdTomato* (*STOP-Tom*) reporter strain that contained the *STOP*-of-transcription sequence that was surrounded by LoxP sites and inserted between the CAG promoter and tdTomato coding sequence. *STOP-Tom;Syn-Cre* double-transgenic mice exhibited strong tdTomato expression in the brain, with the highest fluorescence in the CA3 and dentate gyrus subregions of the hippocampus, midbrain, and cerebellum Purkinje cells but very low expression in the CA1 subregion of the hippocampus and cerebral cortex (S6A Fig). To verify that *Amot* exon 2 was deleted by Cre recombinase in *Amot fl/fl;Syn-Cre* mice, we performed genotyping reactions with genomic DNA that was obtained from the tail and brain. As shown in S6B Fig, the electrophoresis of genotyping reactions revealed a 450-bp PCR fragment that corresponded to the deleted *Amot* sequence for DNA that was obtained from the brain of homozygous mice that expressed Cre (S6B Fig). The western blot analysis of cerebellar and hippocampal homogenates from homozygous mice with the *Syn-Cre* transgene additionally confirmed lower levels of Amot protein (Fig 5B and S6C Fig).

*Amot fl/fl;Syn-Cre* mice were born at the expected mendelian ratio and exhibited no signs of lethality. At birth, the pups of homozygous mice that expressed Cre were significantly smaller and had a lower birth weight compared with their littermate controls of the same sex (S6D, S6E and S6F Fig). These weight differences were apparent until approximately P70. Afterward, *Amot fl/fl;Syn-Cre* mice grew to the same size and weight as *Amot fl/fl mice*, which were similar to wild-type animals (S6F Fig).

### Neuronal deletion of Amot affects cerebellar morphology and the development of dendritic trees of Purkinje cells

We then analyzed the effects of Amot deletion in neurons on brain morphology. We made cryostat sections of brains that were collected from juvenile (P12) and early-adult (P30) animals. *Amot fl/fl;Syn-Cre* mice did not exhibit apparent defects in size or gross morphology of the brain (S7A Fig and Fig 5C). However, the cerebellum appeared to be smaller (Fig 5C) and had a lower weight (Fig 5D) when compared with the cerebellum of littermate controls on P30. The difference in cerebellar weight was also observed in older animals (P150; S7B Fig). Because of this observation and because Syn-Cre activity was more homogeneously distributed in the cerebellum and incomplete in the hippocampus (S6A Fig), we focused our subsequent experiments on the cerebellum. Immunohistochemical studies of sagittal cryosections of the cerebellum revealed that the cerebellar foliation pattern, number of lobes, and number of Purkinje cells were unaffected in the absence of Amot (Fig 5E and 5F and S7C Fig). However, further analysis showed that the molecular layer thickness in lobe III down the preculminate fissure significantly decreased in *Amot* mutant mice compared with control animals (Fig 5G and 5H). The thickness of the molecular layer along the other fissures also decreased, but the differences did not reach statistical significance (S7D Fig). The thickness of the granular and Purkinje cell layers was not significantly affected by Amot deletion (Fig 5I and 5J and S7E and S7F Fig). The distribution of granular cells in the molecular layer in *Amot fl/fl;Syn-Cre* and control mice was also similar (Fig 5K and 5L).

**Fig 6:**
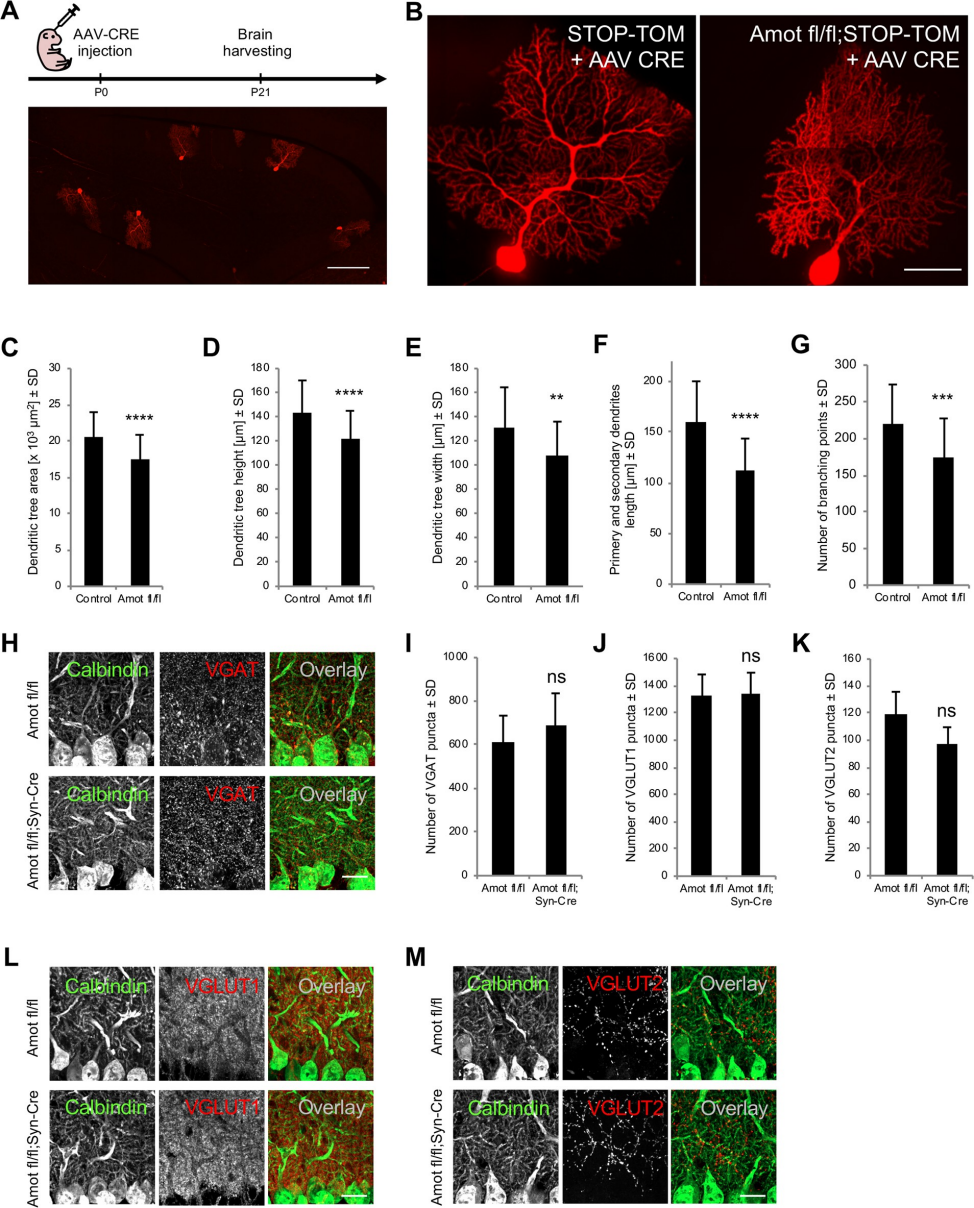
Amot deletion in neurons impairs dendritic tree morphology of Purkinje cells. (A) Tomato expression in single Purkinje cells in *STOP-Tom* reporter mice as a result of Cre activity upon virus injection. The upper panel shows a schematic illustration of the experimental procedure. P0 *STOP-Tom* pups were injected with AAV8-Syn-Cre virus and humanely killed on P21. (B) Images of the dendritic tree morphology of Purkinje cells in *STOP-Tom* and *Amot fl/fl;STOP-Tom* mice that were infected with AAV8-Syn-Cre. (C-G) Quantitative analysis of dendritic tree area (C), height (D), and width (E); primary and secondary dendrite lengths (F); and number of dendritic branching points (G) of Purkinje cells from *STOP-Tom* (*n* ≥ 45 cells and 4 mice in C–F; *n* = 35 cells and 4 mice in G) and *Amot fl/fl;STOP-Tom* (*n* ≥ 43 cells and 4 mice in C–F; *n* = 39 cells and 4 mice in G) brains that were infected with AAV8-Syn-Cre. *p* < 0.0001, *p* < 0.0001, *p* = 0.0012, *p* < 0.0001, and *p* = 0.0007. (H-M) Distribution of inhibitory and excitatory synapses in the cerebellum in *Amot fl/fl* and *Amot fl/fl;Syn-Cre* P30 mice. Cerebellar sagittal sections were immunostained for VGAT (H), VGLUT1 (L), and VGLUT2 (M). Anti-calbindin antibody was used to visualize Purkinje cells. (I-K) Quantification of VGAT (I), VGLUT1 (J), and VGLUT2 (K) puncta per 1,000 μm^2^ in *Amot fl/fl* mice (*n* = 3 mice and at least three sections per mouse) and *Amot fl/fl;Syn-Cre* mice (*n* = 4 mice and at least three sections per mouse). *p* = 0.4891, *p* = 0.9017, and *p* = 0.0970. Numerical values that underlie the graphs are shown in S1 Data. Statistical significance was analyzed using two-tailed unpaired *t* tests. **p < 0.01, ***p < 0.001, ****p < 0.0001. Bars represent the mean ± SD. Scale bars = 200 μm in A, 50 μm in B and 20 μm in H, L, M. Amot, angiomotin; ns, not significant; P, postnatal day; SD, standard deviation; *STOP-Tom*, *STOP-tdTomato*; VGAT, vesicular γ-aminobutyric acid transporter; VGLUT, vesicular glutamate transporter.

To enable visualization of the morphology of individual *Amot*^*−/−*^ Purkinje cells, we intracerebrally injected newborn *Amot fl/fl;STOP-Tom* pups and control *STOP-Tom* pups with low-titer serotype 8 adeno-associated virus (AAV8) that expressed Cre under the neuron-specific synapsin 1 promoter (AAV8-Syn-Cre; Fig 6A). AAV8 has been previously shown to have high tropism to Purkinje cells, allowing the cell type–specific expression of Cre and deletion of Amot in Purkinje cells [44]. Three weeks after viral injection, the animals were humanely killed, and the morphology of Purkinje cell dendritic trees was analyzed using high-magnification images of 100-μm sagittal sections of the cerebellum (Fig 6A and 6B). We measured the dendritic tree height and width, length of primary and secondary branches, dendritic field area, and the number of dendrite branching points of Purkinje cells. To eliminate potential bias, all of the measurements were performed in a blinded manner and confirmed by two independent researchers. *Amot*^*−/−*^ Purkinje cells exhibited significant reductions of all of these measurements compared with control cells, indicating impairments in the dendritic arborization of Purkinje cells in vivo (Fig 6B, 6C, 6D, 6E, 6F and 6G). Amot ablation specifically impaired the complexity of dendritic tree morphology but not the distribution of synaptic markers within the molecular layer, including vesicular γ-aminobutyric acid transporter (VGAT), vesicular glutamate transporter 1 (VGLUT1), and VGLUT2 (Fig 6H, 6I, 6J, 6K, 6L and 6M). This is consistent with a previous study that reported that Amot knockdown in cultured hippocampal neurons did not affect the number of synapses [33].

### Yap1 is required for cerebellar organization and Purkinje cell dendritogenesis

**Fig 7:**
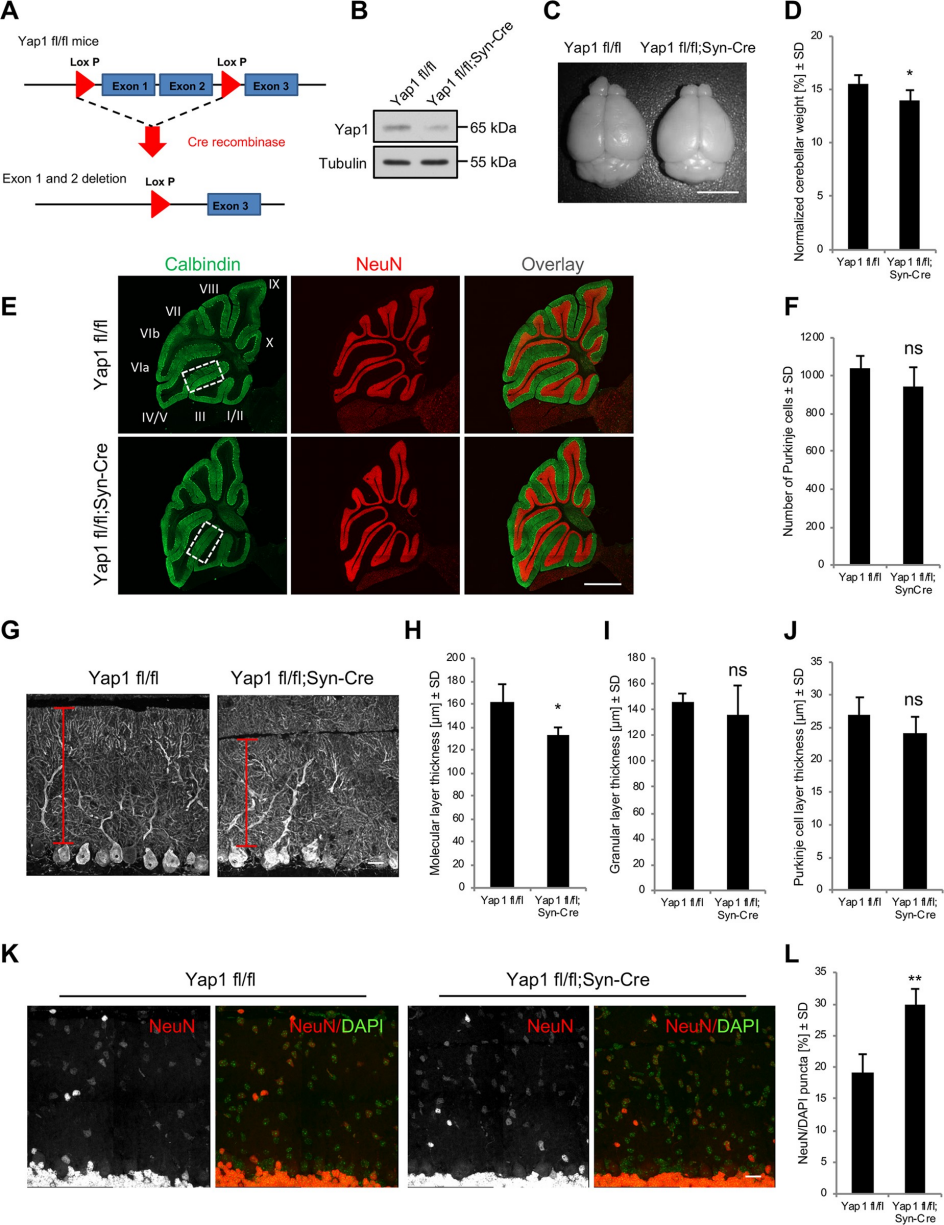
Yap1 deletion in neurons affects cerebellar morphology. (A) Strategy for generating *Yap1* conditional knockout mice, showing the *Yap1 flox* allele (upper diagram) and recombined allele after Cre-mediated excision (lower diagram). (B) Lower Yap1 expression in the cerebellum of *Yap1 fl/fl;Syn-Cre* P30 mice analyzed by western blot. (C) *Yap1 fl/fl;Syn-Cre* mice exhibited a smaller cerebellum compared with control sex-matched littermates (*Yap1 fl/fl*). (D) Quantitative analysis of cerebellar weight in *Yap1 fl/fl* (*n* = 10) and *Yap1 fl/fl;Syn-Cre* (*n* = 4) P30 mice. *p* = 0.0178. Measurements were normalized to whole-brain weight. (E) Sagittal cerebellar sections of *Yap1 fl/fl* and *Yap1 fl/fl;Syn-Cre* P30 mice immunolabeled for calbindin (green) to visualize Purkinje cells and the molecular layer and NeuN (red) to visualize the granule cell layer. Numbers indicate individual lobes. (F) Quantification of Purkinje cells in sagittal sections of *Yap1 fl/fl* mice (*n* = 4 mice and at least three sections per mouse) and *Yap1 fl/fl;Syn-Cre* mice (*n* = 4 mice and at least three sections per mouse). *p* = 0.1782. (G) Decrease in cerebellar molecular layer thickness in *Yap1 fl/fl;Syn-Cre* mice visualized with calbindin; lobe III down the preculminate fissure at P30. (H-J) Thickness of the molecular (H), granular (I), and Purkinje (J) cell layers in the cerebellum of *Yap1 fl/fl* (*n* = 4 mice and at least three sections per mouse) and *Yap1 fl/fl;Syn-Cre* (*n* = 4 mice and at least three sections per mouse) P30 mice measured in lobe III down the preculminate fissure. *p* = 0.0128, *p* = 0.4679, and *p* = 0.1885. (K, L) Granule cell distribution (K) and quantification (L) in the molecular layer in P30 *Yap1 fl/fl* mice (*n* = 4 mice and at least three sections per mouse) and *Yap1 fl/fl;Syn-Cre* mice (*n* = 4 mice and at least three sections per mouse) in the cerebellum, shown as a percentage of NeuN-positive cells per 20,000 μm^2^. *p* = 0.0010. Numerical values that underlie the graphs are shown in S1 Data. Statistical significance was analyzed using two-tailed unpaired *t* test. *p < 0.05, **p < 0.01. Bars represent the mean ± SD. Scale bar = 5 mm in C, 1,000 μm in E, and 20 μm in G and K. ns, not significant; P, postnatal day; SD, standard deviation; Yap1, Yes-associated protein 1.

To investigate the function of Yap1 in vivo, we crossed *Yap1 fl/fl* mice, in which exons 1 and 2 of *Yap1* were flanked by LoxP sites (Fig 7A), with *Syn-Cre* transgenic mice. To verify exon excision, we genotyped genomic DNA that were obtained from the tail and brain. As shown in S8A Fig, the electrophoresis of the genotyping reactions revealed a 697-bp PCR fragment that corresponded to the deleted *Yap1* sequence for DNA that was obtained from the brain of *Yap1 fl/fl;Syn-Cre* (S8A Fig). The western blot analysis of cerebellar homogenates from homozygous mice that expressed Cre additionally confirmed lower levels of Yap1 protein (Fig 7B). *Yap1 fl/fl;Syn-Cre* mice were born according to the mendelian ratio and exhibited no signs of lethality. Homozygous mice that expressed Cre, however, were significantly smaller and had a lower weight compared with their littermate controls of the same sex (S8B and S8C Fig). Similar to *Amot* mutants, *Yap1 fl/fl;Syn-Cre* mice appeared to have a lower cerebellar size (Fig 7C) and cerebellar weight (Fig 7D) compared with littermate controls on P30. The difference in cerebellar weight was also apparent in older animals (P150; S8D Fig). Structural studies of sagittal cryosections of the cerebellum revealed no general abnormalities in cerebellar architecture or the number of Purkinje cells in *Yap1* mutants (Fig 7E and 7F and S8E Fig). Similar to Amot, Yap1 deletion significantly reduced the molecular layer thickness specifically in lobe III down the preculminate fissure (Fig 7G and 7H and S8F Fig). The thickness of the granular and Purkinje cell layers was not significantly affected in *Yap1* mutant mice (Fig 7I and 7J and S8G and S8H Fig). In contrast to Amot, Yap1 deletion increased the number of granular cells in the cerebellar molecular layer (Fig 7K and 7L).

**Fig 8:**
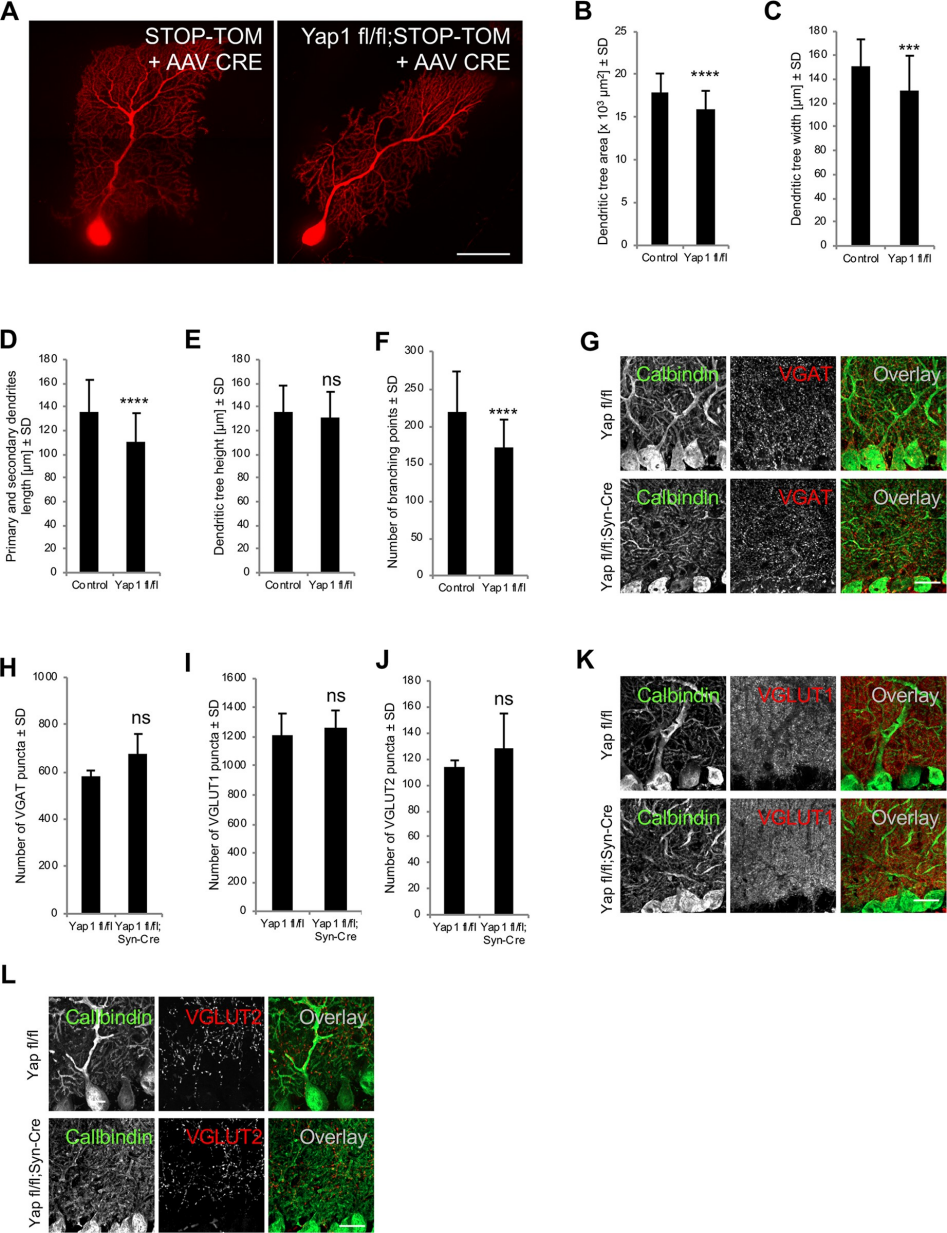
Impairments in dendritic tree morphology of Purkinje cells in Yap1 mutant mice. (A) Dendritic trees of Purkinje cells from *STOP-Tom* (control) and *Yap1 fl/fl;STOP-Tom* mice that were infected with AAV8-Syn-Cre. (B-F) Quantitative analysis of dendritic tree area (B) and width (C), primary and secondary dendrite length (D) and height (E), and the number of dendritic branching points (F) of Purkinje cells from *STOP-Tom* (*n* ≥ 28 cells and 5 mice in B-E; *n* = 33 cells and 5 mice in F) and *Yap1 fl/fl;STOP-Tom* (*n* ≥ 68 cells and 8 mice in B-E; *n* = 48 cells and 6 mice in F) brains infected with AAV8-Syn-Cre. *p* < 0.0001, *p* = 0.0003, *p* < 0.0001, *p* = 0.2194, *p* < 0.0001. (G-L) Distribution of inhibitory and excitatory synapses in the cerebellum in *Yap1 fl/fl* and *Yap1 fl/fl;Syn-Cre* P30 mice. Cerebellar sagittal sections were immunostained for VGAT (G), VGLUT1 (K), and VGLUT2 (L). Anti-calbindin antibody was used to visualize Purkinje cells. (H-J) Quantification of VGAT (H), VGLUT1 (I), and VGLUT2 (J) puncta per 1,000 μm^2^ in *Yap1 fl/fl* mice (*n* = 4 mice and at least three sections per mouse) and *Yap1 fl/fl;Syn-Cre* mice (*n* = 4 mice and at least three sections per mouse). *p* = 0.0727, *p* = 0.6267, and *p* = 0.3417. Numerical values that underlie the graphs are shown in S1 Data. Statistical significance was analyzed using two-tailed unpaired *t* tests. ***p < 0.001, ****p < 0.0001. Bars represent the mean ± SD. Scale bars = 50 μm in A and 50 μm in G, K, and L. ns, not significant; P, postnatal day; SD, standard deviation; *STOP-Tom*, *STOP-tdTomato*; VGAT, vesicular γ-aminobutyric acid transporter; VGLUT, vesicular glutamate transporter; YAP1, Yes-associated protein 1.

Finally, we investigated whether Yap1 also plays a role in the organization of dendritic trees of Purkinje cells. We injected newborn *Yap1 fl/fl;STOP-Tom* mouse pups and control *STOP-Tom* mouse pups with the AAV8-Syn-Cre virus and analyzed the dendritic trees of Purkinje cells. To eliminate potential bias, all of the measurements were performed in a blinded manner and confirmed by two independent researchers. *Yap1*^*−/−*^ Purkinje cells exhibited a significant reduction of dendritic field area, dendritic tree width, the length of primary and secondary branches, and the number of dendrite branching points compared with control cells (Fig 8A, 8B, 8C, 8D and 8F). We did not detect significant differences in dendritic tree height (Fig 8E). Thus, Yap1, similar to Amot, plays an important role in regulating the dendritic arborization of Purkinje cells in vivo. The defect in dendritic tree organization was not associated with significant changes in the distribution of inhibitory (VGAT) or excitatory (VGLUT1 and VGLUT2) synaptic markers within the molecular layer (Fig 8G, 8H, 8I, 8J, 8K and 8L).

### Impairment in motor coordination in mice with neuronal deletion of Amot or Yap1

**Fig 9:**
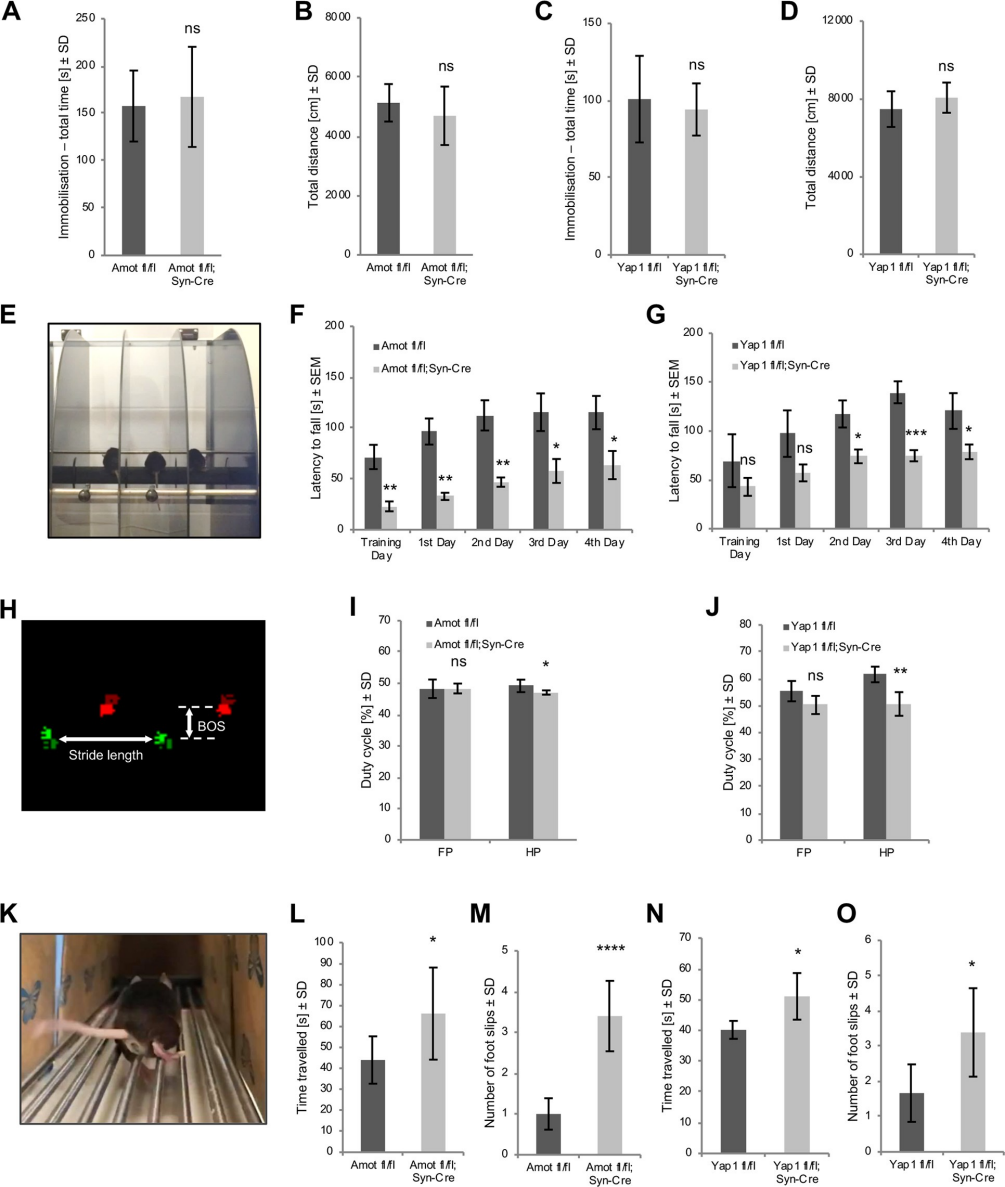
Impairment in locomotor coordination in *Amot fl/fl;Syn-Cre* and *Yap1 fl/fl;Syn-Cre* mice. (A, B) General mobility in *Amot fl/fl;Syn-Cre mice* (*n* = 9) and control age- and sex-matched *Amot fl/fl* mice (*n* = 9) in the open field, showing the (A) time spent immobile (*p* = 0.6602) and (B) total distance traveled (*p* = 0.2787). (C, D) General mobility in *Yap1 fl/fl;Syn-Cre* mice (*n* = 6) and control age- and sex-matched *Yap1 fl/fl* mice (*n* = 8) in the open field, showing the (C) time spent immobile (*p* = 0.5806) and (D) total distance traveled (*p* = 0.2298). (E) Image from the rotarod experiment. (F) Locomotor coordination in *Amot fl/fl* mice (*n* = 10) and *Amot fl/fl;Syn-Cre* mice (*n* = 6) on P150, showing the latency to fall from the rotarod. *p* = 0.0092, *p* = 0.0022, *p* = 0.0051, *p* = 0.0422, and *p* = 0.0483. (G) Locomotor coordination in *Yap1 fl/fl* mice (*n* = 4) and *Yap1 fl/fl;Syn-Cre* mice (*n* = 6) on P150, showing the latency to fall from the rotarod. *p* = 0.3018, *p* = 0.1009, *p* = 0.0150, *p* = 0.0005, and *p* = 0.0416. (H) CatWalk gait analysis. The footprints of the right paws are shown in red, and the footprints of the left paws are shown in green. (I) Duty cycle in *Amot fl/fl;Syn-Cre* mice (*n* = 5) and *Amot fl/fl* (*n* = 11) control littermates. *p* = 0.9463 and *p* = 0.0401. (J) Duty cycle in *Yap1 fl/fl;Syn-Cre* (*n* = 6) and *Yap1 fl/fl* (*n* = 4) mice. *p* = 0.0538 and *p* = 0.0024. (K) Image from the foot-fault experiment. (L, M) Foot-fault analysis of *Amot fl/fl* (*n* = 7) and *Amot fl/fl;Syn-Cre* (*n* = 5) mice. (L) Time traveled. *p* = 0.0441. (M) Number of foot slips. *p* < 0.0001. (N, O) Foot-fault analysis of *Yap1 fl/fl* (*n* = 4) and *Yap1 fl/fl;Syn-Cre* (*n* = 6) mice. (N) Time traveled. *p* = 0.0271. (O) Number of foot slips. *p* = 0.0430. Numerical values that underlie the graphs are shown in S1 Data. Statistical significance was analyzed using two-tailed unpaired *t* test. *p < 0.05, **p < 0.01, ***p < 0.001. Bars represent the mean ± SD in A, B, C, D, I, J, K, M, N, and O and mean ± SEM in F and G. Amot, angiomotin; BOS, base of support; FP, forepaws; HP, hind paws; ns, not significant; P, postnatal day; SD, standard deviation; SEM, standard error of the mean; Yap1, Yes-associated protein 1.

To evaluate whether the defects in cerebellar morphology affected animal mobility, we performed open-field experiments. Amot and Yap1 mutant mice did not differ significantly from control animals in the amount of time spent immobile or distance traveled (Fig 9A, 9B, 9C and 9D and S9G and S9I Fig). In the next set of behavioral experiments, we studied motor coordination that depends on the proper function of the cerebellum. In the first experiment, we placed animals on an accelerating rod and measured the latency to fall (Fig 9E). To minimize differences in body size and weight in younger mice (S6F and S8C Fig), we performed the tests on P150 animals. At this age, both mutants had lower cerebellar weight (S7B and S8D Fig), similar to P30 mice. The latency to fall from a rotating rod in *Amot fl/fl;Syn-Cre* and *Yap1 fl/fl;Syn-Cre* mice was significantly shorter compared with control animals of the same age and sex (Fig 9F and 9G and S9A and S9B Fig). The differences in motor coordination were apparent on the training day and during 4 consecutive days of the test. To study potential gait deficits, we performed a CatWalk experiment, which is an automated analysis of footprints of mice that run across a 130-cm runway (Fig 9H). The mutant mice did not exhibit apparent changes in the base of support (BOS; i.e., the distance between two forepaws or hind paws) (S9C and S9D Fig). *Yap1 fl/fl;Syn-Cre* mice, however, exhibited significant increases in forepaw and hind paw stride measurements, indicating the distance between footsteps of the same paw (S9F Fig). The mean stride length in Amot mutant mice was not significantly affected (S9E Fig). The analysis of step-cycle dynamics revealed that both Amot and Yap1 mutant mice exhibited a significant reduction of the hind paw duty cycle, indicating that the ratio between stance duration and the complete step-cycle duration was smaller compared with control animals (Fig 9I and 9J). Yap1 mutant mice also exhibited a decrease in the duty cycle of the forepaws, but this decrease did not reach statistical significance (Fig 9J). Lastly, we analyzed the performance of mice in the foot-fault test, in which the animals were allowed to walk on a metal rod runway, and the number of footslips from the rods and run time were analyzed based on video recordings (Fig 9K). The performance of Amot and Yap1 mutant mice was significantly worse than control mice. The mutant animals made more footslips and required more time to reach the end of the runway (Fig 9L, 9M, 9N and 9O).

To exclude the potential impact of vestibular system dysfunction on the observed impairments in motor coordination, we performed a more detailed analysis of behavior in the open field. Inner-ear dysfunctions are associated with animal circling behavior [45]. Neither Amot nor Yap1 mutant animals exhibited an increase in the frequency of rotations compared with controls (S9G, S9H, S9I and S9J Fig). These results suggest that the observed behavioral abnormalities were caused by cerebellar impairments.

### Abnormal S6 ribosomal protein phosphorylation in Amot- and Yap1-deficient neurons

In many cell types, the Amot interaction with Yap1 regulates its cotranscriptional activity, resulting in alterations of Hippo pathway–dependent gene expression [18,20,29,32]. To examine whether Amot depletion in neurons affects Yap1-dependent gene expression, we performed qRT-PCR analysis of expression levels of cysteine-rich 61 (*Cyr61*), connective tissue growth factor (*CTGF*), ankyrin repeat domain 1 (*Ankrd1*), cytochrome P450 family 19 subfamily A member 1 (*Cyp19a1*), and amphiregulin (*Areg*). All of these genes are regulated by the Hippo pathway. Amot depletion both in vitro and in vivo did not affect the expression of any of these genes (S10 Fig). This is consistent with our observation that the binding of Yap1 to transcription factors of the TEAD family, which is required for the expression of the Hippo pathway–controlled genes, is not required for dendritic organization (Fig 4C and 4D). Collectively, these results suggest that Yap1 and Amot play a role in dendritogenesis that is independent of interactions with TEAD and TEAD-controlled gene expression.

**Fig 10:**
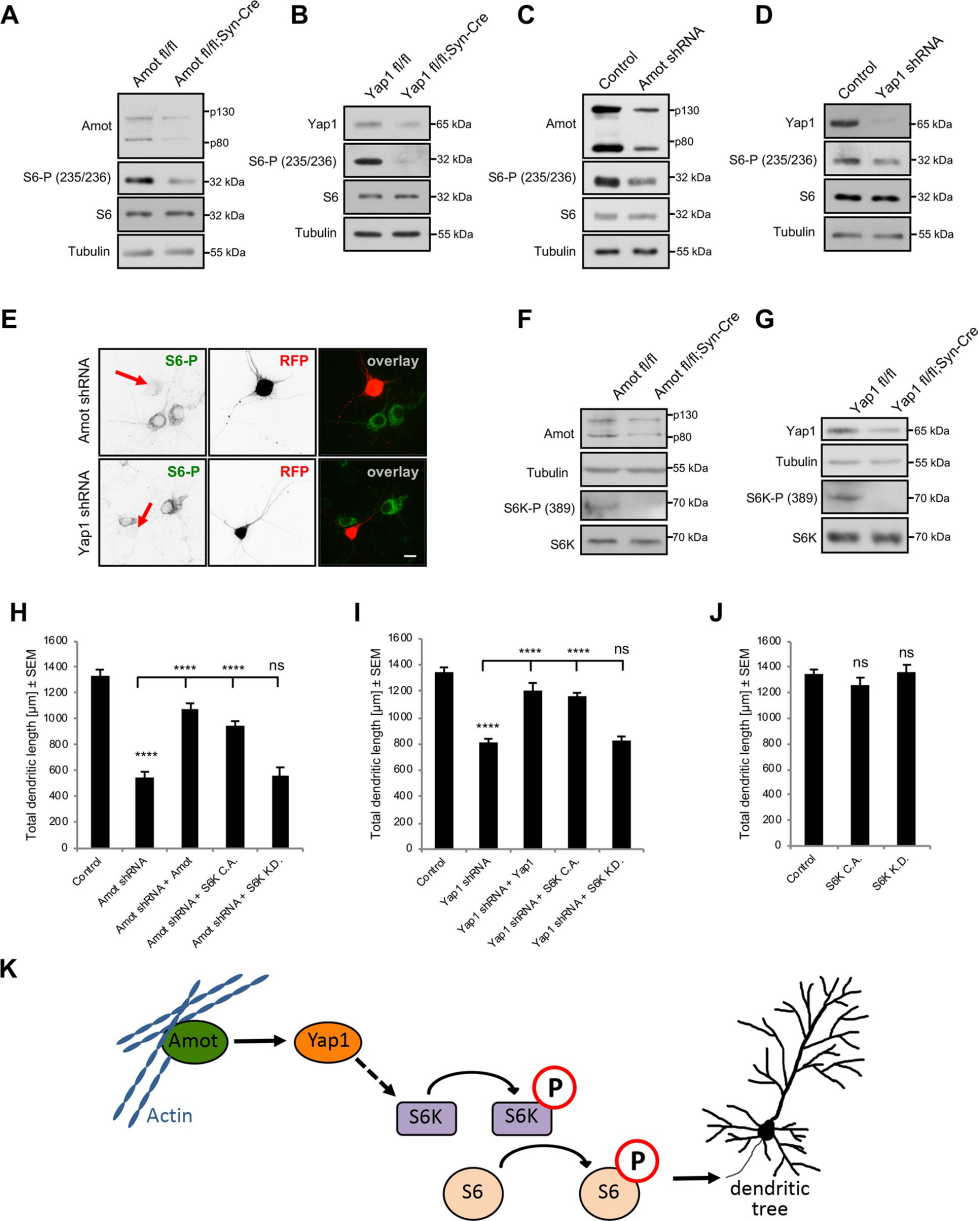
Mechanism underlying Amot- and Yap1-mediated dendritogenesis. (A, B) Reduction of phosphorylation of S6 ribosomal protein in cerebellar homogenates from *Amot fl/fl;Syn-Cre* (A) and *Yap1 fl/fl;Syn-Cre* (B) P30 mice, analyzed by western blot (see also S11A and S11B Fig for additional blots and quantification). (C, D) Reduction of phosphorylation of S6 ribosomal protein levels in lysates from Amot-knockdown neurons (C) and Yap1-knockdown neurons (D), analyzed by western blot (see also S11C Fig for additional blots and quantification). (E) Decrease in phosphorylation of S6 ribosomal protein levels in cultured rat hippocampal neurons on DIV7 transfected with constructs that encoded Amot shRNA or Yap1 shRNA, analyzed by immunocytochemistry (see also S11D for quantification). The neurons were cotransfected with a plasmid that expressed mRFP (red) to visualize transfected cells (arrows in the left panel) and immunostained with anti-phospho-S6 antibody (green). Scale bar = 10 μm. (F, G) Reduction of phosphorylation of p70 S6K at Thr389 in cerebellar homogenates from *Amot fl/fl;Syn-Cre* (F) and *Yap fl/fl;Syn-Cre* (G) P30 mice, analyzed by western blot. (H-J) The ectopic expression of S6K rescued morphological phenotypes in Amot- or Yap1-knockdown neurons. (H) Quantification of TDL of Amot-knockdown rat hippocampal neurons that were transfected with the indicated constructs. Control: *n* = 66; Amot shRNA: *n* = 80; Amot shRNA + Amot: *n* = 48; Amot shRNA + S6K C.A.: *n* = 78; Amot shRNA + S6K K.D.: *n* = 38). To control *p* < 0.0001; to Amot shRNA *p* < 0.0001, *p* < 0.0001, *p* = 0.9995. (I) Quantification of TDL of Yap1-knockdown rat hippocampal neurons that were transfected with the indicated constructs. Control: *n* = 66; Yap1 shRNA: *n* = 92; Yap1 shRNA + Yap1: *n* = 46; Yap1 shRNA + S6K C.A.: *n* = 75; Yap1 shRNA + S6K K.D.: *n* = 46. To control *p* < 0.0001; to Yap1 shRNA *p* < 0.0001, *p* < 0.0001, *p* = 0.9933. (J) Quantification of TDL of rat hippocampal neurons that were transfected with the indicated plasmids. Control: *n* = 66; S6K C.A.: *n* = 37; S6K K.D.: *n* = 38. *p* = 0.5395, *p* = 0.9516. The analysis was performed for neurons from at least three independent cultures. (K) Model of a pathway in which Amot interacts with Yap1 and regulates dendritic tree morphology through the regulation of S6 phosphorylation by S6K. Numerical values that underlie the graphs are shown in S1 Data. Statistical significance was analyzed using one-way analysis of variance followed by Tukey’s post hoc test. ****p < 0.0001. Bars represent the mean ± SEM. Amot, angiomotin; DIV, day in vitro; mRFP, monomeric RFP; ns, not significant; P, postnatal day; RFP, red fluorescent protein; S6K, S6 kinase; S6K C.A., constitutively active mutant of S6K; S6K K.D., kinase-dead mutant of S6K; SEM, standard error of the mean; shRNA, short-hairpin RNA; TDL, total dendrite length; Thr, threonine; Yap1, Yes-associated protein 1.

Recent studies suggested cross talk between angiomotins–Yap1 and the mammalian/mechanistic target of rapamycin (mTOR) signaling pathway [46,47]. Tumaneng and colleagues [47] reported that Yap1 modulates S6K activity, which is a major downstream component of the mTOR pathway that is responsible for the phosphorylation of S6 ribosomal protein. S6K has been reported to regulate dendritic tree organization in neurons [48]. To examine whether Amot and Yap1 influence S6 phosphorylation in neurons, we analyzed the level of S6 phosphorylation at Ser235 and Ser236 that are modified by S6K. The western blot analysis showed that the phosphorylation of S6 at Ser235 and Ser236 in cerebellar homogenates from *Amot fl/fl;Syn-Cre* and *Yap1 fl/fl;Syn-Cre* mice was visibly reduced compared with homogenates from age- and sex-matched control animals (Fig 10A and 10B and S11A and S11B Fig). The levels of total S6 protein remained unchanged (Fig 10A and 10B and S11A and S11B Fig). We observed a similar reduction of S6 phosphorylation in the western blot and immunocytochemistry experiments with cultured neurons that were depleted of Amot and Yap1 (Fig 10C, 10D and 10E and S11C and S11D Fig). Thus, the lack of Amot or Yap1 in neurons was correlated with the reduction of S6 ribosomal protein phosphorylation.

S6K activity is regulated by mTOR-dependent phosphorylation at threonine-389 (Thr389) [49–51]. The levels of phosphorylated S6K significantly decreased in cerebellar homogenates from Amot and Yap1 mutant mice (Fig 10F and 10G). To determine whether the increase in S6 phosphorylation that was induced by S6K (S11E Fig and [52]) could compensate for the defects that were associated with Amot/Yap depletion, we transfected Amot- and Yap1-knockdown neurons with plasmids that expressed a constitutively active mutant of S6K (S6K C.A.) or kinase-dead (inactive) mutant of S6K (S6K K.D.) of S6K. The overexpression of S6K C.A. in Amot-knockdown neurons rescued the morphological defects in dendritic organization to a similar extent as full-length Amot, whereas overexpression of the S6K K.D. mutant failed to rescue the dendritic tree morphology of Amot-depleted neurons (Fig 10H). Similarly, the overexpression of S6K C.A. but not S6K K.D. restored neuronal morphology in Yap1 knockdown cells (Fig 10I). As a control, we transfected wild-type neurons with either S6K C.A. or S6K K.D. However, as reported previously [52], the overexpression of kinase mutants on the wild-type background did not significantly affect the organization of dendritic trees (Fig 10J). Collectively, these findings show that proper S6K activity is important for the organization of dendritic trees and is consistent with previous reports that S6K knockdown significantly affects dendrites in neurons [48]. Our observations suggest that Amot–Yap1 and S6K–S6 function in the same pathway that regulates dendritic trees and that S6K and S6 are downstream of Amot–Yap1 (Fig 10K).

## Discussion

The present study found that Amot distribution changes during neuronal development. At early stages of development, Amot is localized to dendrites, whereas it is concentrated at synapses in mature neurons. Several of our observations demonstrate that Amot plays an important role in dendritic tree organization. The knockdown or knockout of Amot in developing and mature cultured hippocampal neurons led to impairments in dendritic morphology. Conditional knockout animals with Amot deletion in neurons exhibited a significant reduction of the complexity of dendritic trees of Purkinje cells in vivo. We also demonstrated that Amot controls dendritic trees through interactions with its binding partner, Yap1. Similar to Amot, Yap1 was localized to dendritic processes in immature neurons and was relocalized to synapses in fully developed cells. Yap1 coprecipitated with Amot from brain extracts, and the ectopic expression of Amot with mutated Yap1-binding sites failed to rescue phenotypes that were observed in Amot-depleted neurons. The knockdown or knockout of *Yap1* led to dendritic phenotypes that were compared to those in the absence of Amot. Similar to Amot, Yap1 conditional knockout animals exhibited a reduction of the complexity of dendritic trees of Purkinje cells.

Previous studies investigated the involvement of Amot in dendritic spine maturation [33]. In the present study, we found that the loss of Amot also led to impairments in dendritic tree organization, suggesting that Amot may control distinct neuronal processes. The function of Amot in dendritic spine maturation appears to depend on Lats kinase, which phosphorylates Amot within the ID domain. The ID domain has been shown to be required for targeting Amot to postsynaptic machinery, where Amot interacts with Mupp1 and PSD-95 through the C-terminal PDZ-binding motif. The PDZ-binding motif plays an important role in actin turnover in dendritic spines. However, the function of the actin-binding site of Amot in synapse organization has not been investigated. The role of Amot in dendritic tree organization also depends on the ID domain, specifically on Yap1-binding motifs that within the ID domain. The mutation of actin-binding sites in Amot did not significantly affect its role in dendritogenesis. Although the function of Amot in dendritic spine maturation and the regulation of dendritic tree complexity appears to involve different molecular mechanisms, they both underscore the critical role of the Amot ID domain in neurons. One interesting line of investigation would be to examine whether Yap1 is also involved in synapse organization because it is localized to synapses in mature neurons, similar to Amot.

The reduction of dendritic trees of Purkinje cells in Amot and Yap1 mutant mice was associated with a reduction of the thickness of the molecular layer, where dendrites of Purkinje cells are located. Both mutant mice also exhibited a decrease in cerebellar weight and exhibited impairments in locomotor coordination. The overexpression of Yap1 in neurons restored the thickness of the cerebellar molecular layer and locomotor coordination deficits in a mouse model of ataxia [53]. In the present study, the reduction of the thickness of the molecular layer in Amot and Yap1 mutants was the most pronounced in the lobes down the preculminate fissure, which has been proposed to contain leg and foot representations [54,55]. We additionally found that the number of granule cells within the molecular layer in the cerebella in Yap1 mutant mice was significantly affected, a phenotype that was not observed in the Amot mutant. This could indicate that Yap1 may have additional functions (e.g., granule cell migration). This possibility requires further investigation. Such changes in *Yap1 fl/fl;Syn-Cre* mice could explain their stronger phenotype in the footprint pattern gait analysis. An increase in stride length has been previously observed in mice with alterations of granular cell distribution [56].

The observed phenotypes in mice could be even more pronounced if broader Cre-mediated deletion could be achieved. Huang and colleagues [41] reported that *Yap1 fl/fl* mice that expressed Cre under the control of nestin regulatory elements (i.e., expression in progenitors of neural and glial cell lineages) had a substantially smaller brain size, but these authors interpreted this result as a consequence of the loss of Yap1 function solely in glial cells. Our localization and functional experiments, including RNAi-mediated silencing and neuron-specific deletion, suggest that Yap1 is expressed and functions in neurons. This is consistent with previous reports by Okazawa and colleagues [39,40].

The most well-characterized function of Yap1 is that it is a transcriptional coactivator downstream of the Hippo signaling pathway. Upon binding to transcription factors of the TEAD family, Yap1 stimulates the expression of Hippo pathway–dependent genes (e.g., *CTGF* and *Cyr61*). In the present study, however, the ectopic expression of Yap1 with a deleted TEAD-binding region appeared to be able to restore dendrite organization upon Yap1 knockdown (Fig 4C and 4D). Moreover, the deletion of Amot in neurons did not significantly affect the expression of several TEAD target genes (*CTGF*, *Cyr61*, *Ankrd1*, *Cyp19a1*, and *Areg*; S10 Fig). This would suggest that Amot controls dendritic tree complexity independently of a Hippo pathway–controlled gene expression.

The present results suggest that the Amot- and Yap1-dependent regulation of dendritic morphology involves S6 ribosomal protein. The depletion of Amot or Yap1 in cultured neurons or their conditional deletion in mice led to a reduction of S6K phosphorylation and its downstream target S6 ribosomal protein. Moreover, ectopic expression of S6K C.A. rescued the Amot and Yap1 phenotype of dendrite organization.

The exact mechanism by which Amot and Yap1 impinge on S6K activity is unclear. S6K is regulated by mTOR complex 1 (mTORC1), which phosphorylates S6K at position Thr389 [49,51], leading to its activation. Both S6K and mTORC1 are known to regulate dendritic arbor development [48,52,57,58]. The modification of S6K at Thr389 was strongly inhibited in the Amot and Yap1 mutants (Fig 10F and 10G). Accumulating evidence suggests cross talk between angiomotins, Yap1, and mTOR signaling in nonneuronal cells [46,47,59,60]. Amotl2 protein is closely related to Amot and has been shown to interact with mTORC2 [46]. This could affect mTORC1 through Akt, tuberous sclerosis complex 1/2, and Rictor [61–63]. Another mechanism by which Yap1 can affect S6K activity could involve the regulation of transforming growth factor-β (TGF-β)/Smad signaling. Yap1 interacts with Smad7 and inhibits TGF-β receptor activity [64], which in turn regulates protein phosphatase 2 A (PP2A) that subsequently dephosphorylates and inhibits S6K [65]. Yap1 has also been shown to interact with Smad1 and regulate its transcriptional activity and stability in various cell types, including astrocytes [41,66–68].

A link between Amot, Yap1, and S6K signaling in the context of dendritic morphogenesis should be investigated in detail in future studies. This could also shed light on signaling processes that are related to cancer cell biology, cell migration, and proliferation.

## Materials and methods

### Ethics statement

All of the experimental procedures were approved by the First Warsaw Local Ethics Committee for Animal Experimentation (approval no. 633/2018, 270/2017, and 369/2017). The experiments were conducted in accordance with Act on the Protection of Animals Used for Scientific or Educational Purposes (2015). The animals were humanely killed by using isoflurane or decapitation. Mouse newborns were anesthetized by incubation on ice prior to virus injection.

### DNA constructs and antibodies

The β-actin-GFP plasmid (enhanced GFP [eGFP] under the control of β-actin regulatory elements) was described previously [48]. β-actin-mRFP plasmid (mRFP under the control of β-actin regulatory elements) was a gift from Jakub Włodarczyk (Nencki Institute of Experimental Biology, Warsaw, Poland) [69]. Amotp130-delAB was donated by Dannel McCollum (University of Massachusetts Medical School, Worcester, MA, United States) [35]. The following plasmids were obtained from Addgene (Cambridge, MA, USA): pLKO.1 (catalog no. 10878) [70], pEGFP-C3-hYAP1 (catalog no. 17843) [71], pLX304-YAP1_60–89 (catalog no. 59144) [43], HA-AMOT p130 Y242/287A (Amot ΔYap1, catalog no. 32822) [30], and pRK7-HA-S6K1-KR (S6K K.D., catalog no. 8985) [72]. The pRK5-p70S6KT389E (S6K C.A.) plasmid was described previously [52]. pCMV-Flag-Amot was a gift from Chunling Yi (Georgetown University Medical Center, Washington, DC, USA) [31]. The Yap1 and Amot constructs were PCR-amplified and cloned into the β-actin-GFP vector to generate GFP fusion proteins. The pFU-Cherry-TA(3×)-iCre plasmid was a gift from Julie Lefebvre (The Hospital for Sick Children, University of Toronto, Toronto, ON, Canada). The shRNA sequences 5′-GGTGGAGACTGAAATCCAACG-3′ (Amot shRNA) and 5′-GAAACAGCAGGAGTTATTTCG-3′ (Yap1 shRNA) were cloned into the pLKO.1 vector.

The following antibodies were used: anti-α tubulin (Abcam, Cambridge, MA, USA; catalog no. ab18251), anti-Yap1 (Sigma-Aldrich, St. Louis, MO, USA; catalog no. WH0010413M1), anti-Amot (Aviva, San Diego, CA, USA; catalog no. ARP34661_P050), anti-Amot (Santa Cruz Biotechnology, Dallas, TX, USA; catalog no. sc-515262), anti-Map2 (Sigma, St. Louis, MO, USA; catalog no. M1406), anti-Yap1 (Developmental Studies Hybridoma Bank, Iowa City, IA, USA; catalog no. 8J19), anti-phospho-Yap1 (GeneTex, Irvine, CA, USA; catalog no. GTX61793), anti-S6 ribosomal protein (Cell Signaling Technology, Leiden, the Netherlands; catalog no. 2217), anti-phospho-S6 ribosomal protein (Ser235/236; Cell Signaling Technology, Leiden, the Netherlands; catalog no. 4853), anti-synaptophysin 1 (Synaptic Systems, Goettingen, Germany; catalog no. 101004), anti-calbindin (Synaptic Systems, Goettingen, Germany; catalog no. 214006), anti-p70 S6K (Cell Signaling Technology, Leiden, the Netherlands; catalog no. 9202), anti-phospho-p70 S6K (Thr389; Cell Signaling Technology, Leiden, the Netherlands; catalog no. 9206), anti-ankyrin G (NeuroMab, University of California, CA, USA; catalog no. 75–146), anti-NeuN (Abcam, Cambridge, MA, USA; catalog no. ab177487), and anti-VGAT, anti-VGLUT1, and anti-VGLUT2 (Synaptic Systems, Goettingen, Germany; catalog no. 131002, 135302, and 135402, respectively).

Anti-Amot antibody was a gift from Lars Holmgren (Karolinska Institutet, Stockholm, Sweden) [37]. Horseradish peroxidase–conjugated anti-mouse immunoglobulin G (IgG) and anti-rabbit IgG were obtained from Cell Signaling (Danvers, MA, USA; catalog no. 7076 and 7074, respectively). Alexa-Fluor-488-, Alexa-Fluor-568-, and Alexa-Fluor-647-conjugated secondary antibodies were obtained from Thermo Fisher Scientific (Waltham, MA, USA; catalog no. A-11034, A-11004, A-11041, and A-21450). DAPI was purchased from AppliChem (Darmstadt, Germany; catalog no. A4099).

### Cell culture and transfection

Primary hippocampal and cortical neuronal cultures were prepared from Wistar rat and C57BL/6 mouse brains that were collected on embryonic day 18 (E18). The cultures were performed according to the procedure of Banker and Goslin [73]. Neurons were dissociated with trypsin and cultured in Neurobasal medium (Thermo Fisher Scientific, Waltham, MA, USA; catalog no. 21103049) supplemented with 2% B27 (Thermo Fisher Scientific, Waltham, MA, USA; catalog no. 17504044), L-glutamine, and glutamic acid and plated on surfaces coated with poly-D-lysine (Sigma-Aldrich, St. Louis, MO, USA; catalog no. P2636) and laminin (Invitrogen, Grand Island, NY, USA; catalog no. 23017–015). Primary hippocampal neurons were transfected with Lipofectamine 2000 (Thermo Fisher Scientific, Waltham, MA, USA; catalog no. 11668–030) on DIV7 as described previously [74]. For the morphological analysis, neurons were transfected with a plasmid that expressed mRFP to visualize cell morphology and a GFP plasmid (if needed) to maintain the plasmid ratio. The cells were fixed and imaged 3 d after transfection. Amaxa nucleofection was performed with an Amaxa Nucleofector II Device (Lonza Cologne AG, Cologne, Germany; catalog no. AAD-1001S) using the Amaxa Rat Neuron Nucleofector Kit (Lonza Cologne AG, Cologne, Germany; catalog no. VPG-1003) according to the manufacturer’s protocol.

HEK-293 cells (American Type Culture Collection [ATCC], Manassas, VA, USA; catalog no. CRL-1573) were cultured in Dulbecco’s modified Eagle medium (DMEM) that contained 10% fetal bovine serum supplemented with L-glutamine and penicillin-streptomycin. Transient transfections of HEK-293 cells were performed with Lipofectamine 2000 according to the manufacturer’s instructions.

### RNA isolation and qRT-PCR

RNA was isolated using TRIsure reagent (Bioline, Taunton, MA, USA; catalog no. BIO-38033), and cDNA was synthesized using a High-Capacity cDNA Reverse Transcription Kit (Thermo Fisher Scientific, Waltham, MA, USA; catalog no. 4368814) according to the manufacturer’s instructions. qRT-PCR was performed using the Step One Plus Real-Time PCR system (Thermo Fisher Scientific, Waltham, MA, USA) and SYBR Green PCR Master Mix (Thermo Fisher Scientific, Waltham, MA, USA; catalog no. 4309155). The manufacturer’s programs were used for data acquisition, and the results were analyzed using the comparative *Ct* method for relative quantification. The housekeeping gene glyceraldehyde-3-phosphate dehydrogenase (*Gapdh*) was used to standardize the samples. The following primers were used: Gapdh (5′-GGCCTTCCGTGTTCCTAC-3′ and 5′-TGTCATCATACTTGGCAGGTT-3′), Amot (5′-CAGTCATTAGCCACTCTCCTAAC-3′ and 5′-GTCTTAATCCTTCCTTCCATGTC-3′), CTGF (5′-GCGCCTGTTCTAAGACCTGT-3′ and 5′-TGCACTTTTTGCCCTTCTTAATGT-3′), Cyr61 (5′-TGAAGAGGCTTCCTGTCTTTGG-3′ and 5′-AGGACGTAGTCTGAACGATGC-3′), mouse Cyp19a1 (5′-CTTTCAGCCTTTTGGCTTTG-3′ and 5′-ATTTCCACAAGGTGCCTGTC-3′), mouse Ankrd1 (5′-GCTTAGAAGGACACTTGGCGATC-3′ and 5′-GACATCTGCGTTTCCTCCACGA-3′), mouse Areg (5′-GGTCTTAGGCTCAGGCCATTA-3′ and 5′-CGCTTATGGTGGAAACCTCTC-3′), rat Cyp19a1 (5′-CATTTGGACAGGCTGGGTGA-3′ and 5′-GAACTTTCGTCCAGGGGGAT-3′), rat Ankrd1 (5′-AGGGGTTCAGCCACAAGAGG-3′ and 5′-TTTGCCCGTTACCAGCTCCT-3′), and rat Areg (5′-TGGTGACCTGCCATTGTCAT-3′ and 5′-ATGGCTGCTAACGCGATCTT-3′).

### SDS-PAGE and western blot

Cultured cells or brain tissues were lysed with radioimmunoprecipitation assay (RIPA) buffer (50 mM Tris-HCl [pH 8.0], 150 mM NaCl, 2% Igepal CA-630 [NP-40], 0.25% sodium deoxycholate, 1 mM NaF, and 1 mM DTT supplemented with Mini Protease Inhibitor cocktail [Roche, Indianapolis, IN, USA; catalog no. 11873580001]). Proteins were separated by sodium dodecyl sulfate-polyacrylamide gel electrophoresis (SDS-PAGE) and transferred to nitrocellulose membranes (PALL, Port Washington, NY, USA; catalog no. 66485) or polyvinylidene difluoride membranes (Bio-Rad, Hercules, CA, USA; catalog no. 1620174) using the Trans-Blot Turbo system (Bio-Rad, Hercules, CA, USA; catalog no. 1704270). After blocking with 5% milk in Tris-buffered saline with Tween (TBST), primary antibodies were added to the blocking buffer. The membranes were then washed with TBST and incubated with the appropriate horseradish peroxidase–conjugated secondary antibodies. The intensities of protein bands from the western blots were quantified using the Ingenius Bio Imaging system (Syngene, Frederick, MD, USA) and Gene Tools software. The signal intensities from phosphorylated and total proteins were normalized to respective tubulin levels, and the phosphorylated/total protein ratio was calculated.

### Coimmunoprecipitation

Mouse brain tissue was homogenized in ice-cold lysis buffer (50 mM Tris-HCL [pH 8], 150 mM NaCl, 0.5% Igepal CA-630 [NP-40], 10% glycerol, 1 mM DTT, 1 mM NaF, and Mini Protease Inhibitor cocktail [Roche, Indianapolis, IN, USA; catalog no. 11873580001]). The lysate was then passed three times through a 25-gauge needle with a syringe, rotated at 4°C for 15 min, and centrifuged for 30 min at 18,000*g* at 4°C. After centrifugation, the supernatant was incubated overnight with Dyneabeads Protein G (Thermo Fisher Scientific, Waltham, MA, USA; catalog no. 10004D) coated with anti-Yap1 antibody (Sigma-Aldrich, St. Louis, MO, USA; catalog no. WH0010413M1). Beads with attached proteins were washed four times with lysis buffer, resuspended in 2× sample buffer, and boiled for 10 min. For western blot, samples were subjected to SDS-PAGE.

### Immunocytochemistry and immunohistochemistry

Hippocampal neurons were grown on glass coverslips, fixed with 4% paraformaldehyde (PFA), and blocked with blocking buffer (2% bovine serum albumin [BSA], 2% normal goat serum, and 0.5% Triton X-100 in PBS). The primary and secondary antibodies were applied in blocking buffer.

For brain immunohistochemistry, the mice were transcardially perfused with ice-cold PBS, followed by perfusion with 4% PFA in PBS. The brains were removed, postfixed in 4% PFA overnight at 4°C, and cryoprotected in 30% sucrose. Coronal brain slices (40 μm) were obtained with a cryostat (Leica 1950 Ag Protect; Leica, Wetzlar, Germany) and collected in antifreeze solution (30% ethylene glycol, 15% sucrose, and 0.05% sodium azide in PBS). After incubation in blocking buffer (10% BSA and 0.3% Triton X in PBS), primary antibodies that were diluted in blocking buffer were applied. Fluorophore-conjugated secondary antibodies were diluted in 0.3% Triton X in PBS.

### Mouse strains and husbandry

All of the mice were on a C57BL/6 genetic background and bred in a specific pathogen–free (SPF) animal facility at the Nencki Institute of Experimental Biology. *Amot fl/fl;Syn-Cre* and *Yap1 fl/fl*,*Syn-Cre* mice were generated by crossing *Amot fl/fl* [31] and *Yap1 fl/fl* mice [75], respectively, with *Syn-CRE* transgenic mice (B6.Cg-Tg[Syn1-cre]671Jxm/J). *Syn-CRE* and *Yap1 fl/fl* mice (*Yap1*^*tm1*.*1Dupa*^/J) were obtained from Jackson Laboratory (Bar Harbor, ME, USA; catalog no. 003966 and 027929, respectively). For single Purkinje cell analysis, *Amot fl/fl* and *Yap fl/fl* mice were crossed with the *STOP-Tom* reporter strain [76]. All of the experimental procedures were approved by the First Warsaw Local Ethics Committee for Animal Experimentation, and the experiments were conducted in accordance with all applicable laws and regulations. The following primers were used for genotyping: Amot (5’-GATGGATGCTATGAGAAGGTG-3′ and 5′-GTAAGGATTACAGAGTCTGGG-3′; wild type, approximately 400 bp; flox, approximately 500 bp; conditional knockout, no fragments), Amot (5′-ATAGCTAGTGAGCAGTAGCAG-3′ and 5′-GTAAGGATTACAGAGTCTGGG-3′; wild type, approximately 1 kbp; flox, approximately 1.2 kbp; conditional knockout, approximately 400 bp), and Yap1 (5′-CCATTTGTCCTCATCTCTTACTAAC-3′ and 5′-GATTGGGCACTGTCAATTAATGGGCTT-3′ and 5′-CAGTCTGTAACAACCAGTCAGGGATAC-3′; wild type, approximately 498 bp; flox, approximately 597 bp; conditional knockout, approximately 697 bp).

### Viral infection

Newborn pups (P0) were intracerebrally injected with 1.7 × 10^9^ viral genomes (VGs) of AAV8-Syn-Cre (SignaGen Laboratories, Rockville, MD, USA; catalog no. SL100883) as described previously [44]. Three weeks after viral infection, the animals were intracardially perfused with 4% PFA as described above, and brains were harvested and sectioned into 100-μm coronal slices.

### Behavioral tests

For the open-field test, each mouse was monitored in a round Plexiglas box (64-cm diameter) that was enclosed by 30-cm-high walls using an overhead video tracking system that was connected to EthoVision (Noldus Information Technology, Wageningen, the Netherlands). The animals were taken to the behavioral examination room 1 h before the measurements. Each animal was placed in the middle of the arena, and its movements were recorded for 10 consecutive minutes. The arena was cleaned after each animal with a 70% ethanol solution and dried. The videos were collected in digital format and analyzed using a video tracking system (EthoVision, Noldus, Wageningen, the Netherlands) to extract the behavioral data, including motor measurements (distance traveled and time spent immobile).

To assess motor coordination, a computer-interfaced rotarod that accelerated from 4 to 40 rotations per minute over 240 s was used (TSE, Bad Homburg, Germany). The diameter of the rod was 30 mm. The animals were taken to the behavioral examination room 1 h before the measurements on consecutive days to allow habituation. Habituation began 1 d before the experiment. The animals were allowed to stand on the rotarod for 10–20 s before it began to rotate. The mice were tested in three trials per day, with a 20-min intertrial interval, over 3 consecutive days. The time that each mouse maintained its balance on the rotating rod was measured as the latency to fall.

Gait analysis was performed using CatWalk 7.1 (Noldus Information Technology, Wageningen, the Netherlands), a video-based, digital gait analysis system. Briefly, mice traversed a 130-cm-long glass plate that was parallel to the floor with a fluorescent tube that was connected alongside one of the longer edges. Light from an encased fluorescent bulb was internally reflected within the glass walkway and scattered when the plantar surface of the paw contacted the walkway floor, thereby producing paw prints. The paw prints were recorded by a high-speed camera that was mounted below the walkway and processed using CatWalk 7.1 acquisition software. The test was performed in a darkened room. Three uninterrupted runs were collected for each animal that had been habituated to the experimental conditions and pretrained to run along runway the day before recording. The paw print designations and data analysis were performed by an experimenter who as blinded to the animal genotypes.

The foot-fault test was performed on parallel stainless steel bars (50-cm length, 12-mm diameter) that were placed 15 mm apart. Each mouse was placed at the beginning of a platform and allowed to freely move toward a self-made mouse shelter that was localized at the end of a 50-cm-long runway. Three runs were recorded for each animal, and the total number of footslips and time to traverse the runway were measured for each run.

### Confocal microscopy, image analysis, and quantification

Fluorescence microscopy was performed at the Confocal Microscopy Facility, Nencki Institute, using a spinning disc confocal microscope (Zeiss, Jena, Germany) equipped with PLN APO 10×/0.45, C APO 40×/1.20, and PLN APO 63×/1.40 objectives or Zeiss LSM800 Airyscan (Zeiss, Jena, Germany) equipped with a PLN APO 63×/1.40 objective and Airyscan Detector (32× GaAsP detectors). Images were collected using ZEN software (Zeiss, Jena, Germany) and analyzed using ImageJ software [77].

The morphometric analysis and quantification of dendritic tree complexity were performed using ImageJ software with the Neuronal Tracer plug-in to calculate the TDL and Sholl plug-in for the Sholl analysis. Briefly, each image was processed with the Neuronal Tracer plug-in, and a mask of all dendrites on a confocal image was drawn manually. To avoid background that could be present in the original pictures, the mask was saved as a separate image and used in the automated Sholl analysis. Dendrites and axons were primarily distinguished by morphological criteria as previously described [78]. “Short” neurites with decreasing diameter, splitting with narrow angles (usually <90°) were considered dendrites, and “long” neurites with a relatively constant diameter splitting with variable angles (often ≥90°) were considered axons. The additional criterion for discrimination between dendrites and axons was the presence of dendritic filopodia and dendritic spines. The accuracy of morphology-based classification was additionally confirmed by immunofluorescent staining for Map2 (marker of dendrites) and ankyrin G (marker of axon initial segment). For the Purkinje cell analysis, confocal Z-stacks were collected, and orthogonal projections were generated. Purkinje cell dendritic arbor area, width, height, and length were manually traced using ImageJ software. Primary dendrites of Purkinje cells were defined as branches that emerged from the soma. Secondary dendrites were defined as branches that emerged from primary dendrites with a diameter not smaller than half of the primary dendrite from which they protruded. To eliminate potential bias, all of the measurements were performed in a blinded manner and confirmed by two independent researchers.

### Statistical analysis

Quantitative data are expressed as the mean ± SEM or SD. The statistical methods (two-tailed and unpaired *t* tests, one-way analysis of variance followed by Tukey’s post hoc test, and two-way analysis of variance followed by Bonferroni’s post hoc test) and *p*-values are defined in the figure legends. All of the observations and analyses were performed based on at least three independent experiments. The quantitative analyses of Purkinje cell morphology, rotarod test data, and cerebellar weight data were performed in a blinded manner and confirmed by two independent researchers. Animals were assigned to experimental groups based on their genotypes. The statistical analyses were performed using GraphPad Prism 7 and Microsoft Excel software.

## Supporting information

S1 Fig Localization of Amot–GFP in cultured neurons and specificity of anti-Amot and anti-Yap1 antibodies (related to Figs 1 and 3 in main text). (A) Rat DIV10 hippocampal neurons that expressed Amot–GFP. (B) Cerebellar cross-sections of control *Amot fl/fl* and *Amot fl/fl;Syn-Cre* mice stained for Amot (red; the same image acquisition settings) and calbindin (green). (C) Cerebellar cross-sections of control *Yap1 fl/fl* and *Yap1 fl/fl;Syn-Cre* mice stained for Yap1 (red; the same image acquisition settings) and calbindin (green). Scale bar = 10 μm in A and 20 μm in B and C. Amot, angiomotin; DIV, day in vitro; GFP, green fluorescent protein; Yap1, Yes-associated protein 1.
(TIF)

S2 Fig Cre expression in cultured neurons does not affect dendritic growth or Amot or Yap1 expression levels (related to Figs 2 and 4 in main text). (A) Expression and nuclear localization of Cre in cultured neurons. Representative images of *Amot fl/fl* mouse hippocampal neurons that were cotransfected with a plasmid that expressed Cre–RFP and a vector with GFP that was used to visualize neuronal morphology. (B) Western blot analysis of Amot expression levels in wild-type mouse cortical neurons that were nucleofected with a control or Cre-expressing plasmid. (C) Representative images of cultured wild-type mouse hippocampal neurons that were transfected with a plasmid that encoded Cre recombinase or a control vector. Scale bars = 100 μm. (D) Quantification of TDL of wild-type mouse hippocampal neurons that were transfected with plasmids that encoded Cre recombinase (*n* = 46) or a control vector (*n* = 39). The values are shown as percentage of Control. *p* = 0.7519. The cells were additionally transfected with a GFP vector to visualize neuronal morphology. Quantification was performed for samples that were obtained from at least three independent cultures. (E) Western blot analysis of Yap1 expression levels in wild-type mouse cortical neurons that were nucleofected with a control or Cre-expressing plasmid. (F) Quantification of TDL of mature rat hippocampal neurons that were depleted of Amot and Yap1. The cells were additionally transfected with a GFP vector to visualize neuronal morphology. The cells were transfected with the indicated plasmids on DIV14 and fixed 4 d later. Control: *n* = 69; Amot shRNA: *n* = 60; Yap1 shRNA: *n* = 37. To Control *p* < 0.0001, *p* = 0.0005. Quantification was performed on samples that were obtained from at least three independent cultures. Scale bars = 50 μm. Numerical values that underlie the graph are shown in S1 Data. Statistical significance was analyzed using two-tailed unpaired *t* tests (D) and one-way analysis of variance followed by Tukey’s post hoc test (F). ***p < 0.001, ****p < 0.0001. Bars represent the mean ± SEM. Amot, angiomotin; DIV, day in vitro; GFP, green fluorescent protein; ns, not significant; RFP, red fluorescent protein; SEM, standard error of the mean; TDL, total dendrite length; Yap1, Yes-associated protein 1.
(TIF)

S3 Fig Amot deletion in cultured neurons does not affect neuronal polarization (related to Fig 2 in main text). (A, B) Representative images of *Amot fl/fl* mouse hippocampal neurons that were cotransfected with a plasmid that expressed Cre–RFP or a control vector that were immunolabeled for Map2 (A) or ankyrin G (B). (C) *Amot fl/fl* hippocampal neurons that were cotransfected with a plasmid that expressed Cre–RFP (*n* = 33) or a control vector (*n* = 40), classified according to the number of axons: no axon, single axon, or multiple axons. The cells were cotransfected with a vector that expressed GFP to visualize neuronal morphology. Quantification was performed from at least three independent cultures. Numerical values that underlie the graph are shown in S1 Data. Scale bars = 50 μm. Amot, angiomotin; GFP, green fluorescent protein; Map2, microtubule-associated protein 2; RFP, red fluorescent protein.
(TIF)

S4 Fig Expression levels and localization of Amot and Yap1 constructs in cultured hippocampal neurons (related to Figs 2–4 in main text). (A-C) Extracts from rat neurons that were cotransfected with plasmids that expressed the indicated constructs were analyzed by western blot using anti-GFP antibody. (D, E) Rat DIV10 hippocampal neurons that expressed the indicated Amot and Yap1 constructs. Scale bars = 10 μm. See Results section for further details. Amot, angiomotin; DIV, day in vitro; GFP, green fluorescent protein; Yap1, Yes-associated protein 1.
(TIF)

S5 Fig Yap1 deletion in cultured neurons does not affect neuronal polarization (related to Fig 4 in main text). (A, B) Representative images of *Yap1 fl/fl* mouse hippocampal neurons that were cotransfected with a plasmid that expressed Cre–RFP or a control vector and immunolabeled for Map2 (A) or ankyrin G (B). (C) *Yap1 fl/fl* hippocampal neurons that were cotransfected with a plasmid that expressed Cre–RFP (*n* = 53) or a control vector (*n* = 53), classified according to the number of axons: no axon, single axon, or multiple axons. The cells were cotransfected with a vector that expressed GFP to visualize neuronal morphology. Quantification was performed from at least three independent cultures. Numerical values that underlie the graph are shown in S1 Data. Scale bars = 50 μm. GFP, green fluorescent protein; Map2, microtubule-associated protein 2; RFP, red fluorescent protein; Yap1, Yes-associated protein 1.
(TIF)

S6 Fig Characterization of mice with neuronal deletion of Amot (related to Fig 5 in main text). (A) Cross-sections of brains from *STOP-Tom;Syn-Cre* P30 mice showed high Cre activity in the cerebellum (lower panel) and hippocampus (upper panel). DAPI (gray) was used to visualize brain structures. Scale bars = 1 mm. The insets show higher-magnification images of the hippocampus (upper panel, scale bar = 500 μm) and Purkinje cells (lower panel, scale bar = 50 μm). (B) Confirmation of *Amot* exon2 excision in the brain of *Amot fl/fl;Syn-Cre* mice, assessed by PCR analysis of genomic DNA that was obtained from the tail or brain. (C) Reduction of Amot expression in the hippocampus of *Amot fl/fl;Syn-Cre* P30 mice, analyzed by western blot. (D, E) Images of *Amot fl/fl;Syn-Cre* and *Amot fl/fl* control littermates at the neonatal stage (D) or on P30 (E). Scale bars = 1 cm in C and D. (F) Weight analysis of *Amot fl/fl* and *Amot fl/fl;Syn-Cre* mice (*n* = 8, 9, 7, 3, 4, and 4, and *n* = 6, 4, 9, 6, 6, and 7, respectively) on the indicated days of development. The values are shown as a percentage of control *Amot fl/fl* mouse weights at the corresponding age. *p* < 0.0001, *p* < 0.0001, *p* < 0.0001, *p* = 0.0048, *p* = 0.0561, and *p* = 0.7799. Numerical values that underlie the graphs are shown in S1 Data. Statistical significance was analyzed using two-tailed unpaired *t* test. **p < 0.01, ****p < 0.0001. Bars represent the mean ± standard deviation (SD). Amot, angiomotin; *fl/fl*, *Amot* homozygote mice; ns, not significant; P, postnatal day; SD, standard deviation; WT, wild-type control; *WT/fl*, *Amot* heterozygote mice.
(TIF)

S7 Fig Alteration of cerebellar morphology in *Amot fl/fl;Syn-Cre mice* (related to Fig 5 in main text). (A) Coronal sections of *Amot fl/fl* and *Amot fl/fl;Syn-Cre* brains on P12 and P30 that were stained with DAPI. Scale bars = 1 mm. (B) Quantitative analysis of cerebellar weight of *Amot fl/fl* (*n* = 7) and *Amot fl/fl;Syn-Cre* (*n* = 3) P150 mice normalized to the whole-brain weight. *p* < 0.0001. (C) Quantification of Purkinje cells in individual lobes of the cerebellum of *Amot fl/fl* (*n* = 3 mice and at least three sections per mouse) and *Amot fl/fl;Syn-Cre* (*n* = 5 mice and at least three sections per mouse) P30 mice. Numbers indicate the lobes. *p* = 0.3669, *p* = 0.2006, *p* = 0.4707, *p* = 0.5413, *p* = 0.6975, *p* = 0.1447, *p* = 0.4154, *p* = 0.5165. (D-F) Thickness of the molecular (D), granular (E), and Purkinje (F) cell layers in the cerebellum of *Amot fl/fl* (*n* = 3 mice and at least three sections per mouse) and *Amot fl/fl;Syn-Cre* (*n* = 5 mice and at least three sections per mouse) P30 mice measured at the preculminate, primary, and secondary fissures. *p* = 0.0135, *p* = 0.6818, and *p* = 0.0859 in D; *p* = 0.4169, *p* = 0.9693, and *p* = 0.4702 in E; *p* = 0.4615, *p* = 0.3473, and *p* = 0.5603 in F. Numerical values that underlie the graphs are shown in S1 Data. Statistical significance was analyzed using two-tailed unpaired *t* test. *p < 0.05, ****p < 0.0001. Bars represent the mean ± SD. ns, not significant; P, postnatal day; SD, standard deviation.
(TIF)

S8 Fig Characterization of mice with neuronal deletion of Yap1 (related to Fig 7 in main text). (A) Confirmation of *Yap1* exon1–2 excision in the brain of *Yap1 fl/fl;Syn-Cre* mice, assessed by PCR analysis of genomic DNA that were obtained from the tail or brain. (B) *Yap1 fl/fl;Syn-Cre* P30 mice appeared to be smaller than *Yap1 fl/fl* control littermates. (C) Weight analysis of *Yap1 fl/fl* (*n* = 20) and *Yap1 fl/fl;Syn-Cre* (*n* = 9) P30 mice. *p* = 0.0036. (D) Quantitative analysis of cerebellar weight of *Yap1 fl/fl* (*n* = 7) and *Yap1 fl/fl;Syn-Cre* (*n* = 9) P150 mice normalized to the whole-brain weight. *p* = 0.0023. (E) Quantification of Purkinje cells in individual lobes of *Yap1 fl/fl* (*n* = 4 mice and at least three sections per mouse) and *Yap1 fl/fl;Syn-Cre* (*n* = 4 mice and at least three sections per mouse) P30 mice. Numbers indicate the lobes. *p* = 0.9206, *p* = 0.6583, *p* = 0.4626, *p* = 0.0574, *p* = 0.1038, *p* = 0.1003, *p* = 0.8263, *p* = 0.2776. (F-H) Thickness of the molecular (F), granular (G) and Purkinje (H) cell layers in the cerebellum of *Yap1 fl/fl* (*n* = 4 mice and at least three sections per mouse) and *Yap1 fl/fl;Syn-Cre* (*n* = 4 mice and at least three sections per mouse) P30 mice measured at the preculminate, primary, and secondary fissures. *p* = 0.0128, *p* = 0.2086, and *p* = 0.7616 in F; *p* = 0.4679, *p* = 0.5387, and *p* = 0.6736 in G; *p* = 0.1885, *p* = 0.3746, and *p* = 0.6526 in H. Numerical values that underlie the graphs are shown in S1 Data. Statistical significance was analyzed using two-tailed unpaired *t* test. *p < 0.05, **p < 0.01. Bars represent the mean ± SD. *fl/fl*, *Yap1* homozygote mice; ns, not significant; P, postnatal day; SD, standard deviation; WT, wild-type control; *WT/fl*, *Yap1* heterozygote mice; Yap1, Yes-associated protein 1.
(TIF)

S9 Fig Behavioral analysis of Amot and Yap1 mutants (related to Fig 9 in main text). (A) Locomotor coordination in *Amot fl/fl* (*n* = 5) and *Amot fl/fl;Syn-Cre* (*n* = 5) P100 mice, reflected by the latency to fall from the rotarod. *p* = 0.0988, *p* = 0.0029, and *p* = 0.0137. (B) Locomotor coordination in *Yap1 fl/fl* (*n* = 5) and *Yap1 fl/fl;Syn-Cre* (*n* = 5) P80 mice, reflected by the latency to fall from the rotarod. *p* = 0.2611, *p* = 0.0270, and *p* = 0.0138. (C) CatWalk gait analysis of BOS in Amot mutant mice (*Amot fl/fl*: *n* = 11; *Amot fl/fl;Syn-Cre*: *n* = 5; *p* = 0.2418 and *p* = 0.2081) and (D) CatWalk gait analysis of BOS in Yap1 mutant mice (*Yap1 fl/fl*: *n* = 4; *Yap1 fl/fl;Syn-Cre*, *n* = 6; *p* = 0.2571 and *p* = 0.2243). (E) CatWalk gait analysis of stride length in Amot mutant mice (*Amot fl/fl*: *n* = 11; *Amot fl/fl;Syn-Cre*: *n* = 5; *p* = 0.0922 and *p* = 0.2643). (F) CatWalk gait analysis of stride length in Yap1 mutant mice (*Yap1 fl/fl*: *n* = 4; *Yap1 fl/fl;Syn-Cre*: *n* = 6; *p* = 0.0013 and *p* = 0.0030). (G-J) Open-field analysis of Amot (G, H) and Yap1 (I, J) mutant mice (*Amot fl/fl;Syn-Cre*: *n* = 9; *Amot fl/fl*: *n* = 9; *Yap1 fl/fl;Syn-Cre*: *n* = 6; *Yap1 fl/fl*: *n* = 8). *p* = 0.3930 in H and *p* = 0.4220 in J. Numerical values that underlie the graphs are shown in S1 Data. Statistical significance was analyzed using two-tailed unpaired *t* tests. *p < 0.05, **p < 0.01. Bars represent the mean ± SD. Amot, angiomotin; BOS, base of support; FP, forepaws; HP, hind paws; ns, not significant; P, postnatal day; SD, standard deviation; Yap1, Yes-associated protein 1.
(TIF)

S10 Fig Amot loss of function does not alter hippocampal pathway–dependent gene expression in neurons (related to Fig 10 in main text). (A-J) qRT-PCR analysis of Cyr61 (A, F), CTGF (B, G), Ankrd1 (C, H), Cyp19a1 (D, I), and Areg (E, J) expression in rat cortical neurons (A-E) with Amot knockdown (*n* = 3/group) and cerebellar homogenates (F-J) of Amot knockouts (*n* = 6 in F and G; *n* = 4 in H, I and J) or control P30 mice (*n* = 4 in F and G; *n* = 6 in H, I and J). *p* = 0.0599, *p* = 0.3478, *p* = 0.1014, *p* = 0.4786, *p* = 0.8153, *p* = 0.057, *p* = 0.9804, *p* = 0.6133, *p* = 0.7081, *p* = 0.6600. Numerical values that underlie the graphs are shown in S1 Data. Statistical significance was analyzed using two-tailed unpaired *t* tests. Bars represent the mean ± SEM. Amot, angiomotin; Ankrd1, ankyrin repeat domain 1; Areg, amphiregulin; CTGF, connective tissue growth factor; Cyp19a1, cytochrome P450 family 19 subfamily A member 1; Cyr61, cysteine-rich 61; ns, not significant; P, postnatal day; qRT-PCR, quantitative real-time polymerase chain reaction; SEM, standard error of the mean.
(TIF)

S11 Fig S6 protein phosphorylation in the absence of Amot and Yap1 (related to Fig 10 in main text). (A-C) Western blot analysis and quantification of S6 phosphorylation at Ser235/236 in cerebellum homogenates of *Amot fl/fl* and *Amot fl/fl;Syn-Cre* mice (A) and *Yap1 fl/fl* and *Yap1 fl/fl;Syn-Cre* mice (B) and in rat cortical neurons that were nucleofected with Amot shRNA, Yap1 shRNA, or control plasmid (C). *p* < 0.0001, *p* = 0.0002, *p* = 0.0155, *p* = 0.0147. Tubulin is shown as a loading control. The signal was normalized to the control. (D) Quantification of phosphorylated S6 (Ser235/236) fluorescence intensity in cultured hippocampal neurons that were transfected with control plasmid (*n* = 63), Amot shRNA (*n* = 42), and Yap1 shRNA (*n* = 41). *p* = 0.0005 and *p* < 0.0001. (E) Ectopic expression of S6K C.A. but not S6K K.D. led to an increase in S6 phosphorylation at Ser235/236 in HEK-293 cells. Numerical values that underlie the graphs are shown in S1 Data. Statistical significance was analyzed using two-tailed unpaired *t* tests. *p < 0.05, ***p < 0.001, and ****p < 0.001. Bars represent the mean ± SD. Amot, angiomotin; HEK-293, human embryonic kidney 293; NT, not transfected; S6K C.A., constitutively active S6 kinase; S6K K.D., kinase-dead (inactive) mutant S6 kinase; SD, standard deviation; Ser, serine; shRNA, short-hairpin RNA; Yap1, Yes-associated protein 1.
(TIF)

S1 Table Statistical significance of Sholl analysis plots (related to Figs 2 and 4 in main text). Sholl analysis was performed for hippocampal neurons that were transfected with the indicated plasmids at distances 10–200 μm from the cell body. Significance for particular radii was assessed using two-way analysis of variance followed by Bonferroni’s post hoc test. The *p*-values are indicated in the table.
(XLSX)

S1 Data All individual numerical values that underlie the summary data that are shown in Figs 2A, 2D, 2E, 2G, 2H, 2K, 3C, 4C, 4D, 4G, 4H, 5D, 5F, 5H, 5I, 5J, 5L, 6C, 6D, 6E, 6F, 6G, 6I, 6J, 6K, 7D, 7F, 7H, 7I, 7J, 7L, 8B, 8C, 8D, 8E, 8F, 8H, 8I, 8J, 9A, 9B, 9C, 9D, 9F, 9G, 9I, 9J, 9L, 9M, 9N, 9O, 10H, 10I, 10J, S2D, S2F, S3C, S5C, S6F, S7B, S7C, S7D, S7E, S7F, S8C, S8D, S8E, S8F, S8G, S8H, S9A, S9B, S9C, S9D, S9E, S9F, S9H, S9J, S10A, S10B, S10C, S10D, S10E, S10F, S10G, S10H, S10I, S10J, S11A, S11B, S11C and S11D. (XLSX)

## Abbreviations

AAV8: serotype 8 adeno-associated virus
Amot: angiomotin
Amotl1: angiomotin-like 1
Amotl2: angiomotin-like 2
Ankrd1: ankyrin repeat domain 1
Areg: amphiregulin
BOS: base of support
CC: coiled-coil domain
CTGF: connective tissue growth factor
Cyp19a1: cytochrome P450 family 19 subfamily A member 1
Cyr61: cysteine-rich 61
DIV: day in vitro
GFP: green fluorescent protein
HEK-293: human embryonic kidney 293
ID: intrinsic disordered
Lats: large tumor suppressor kinase
Map2: microtubule-associated protein 2
mRFP: monomeric red fluorescent protein
Mst: mammalian sterile 20-like kinase
mTOR: mammalian/mechanistic target of rapamycin
mTORC1: mTOR complex 1
MUPP1: multi-PDZ domain protein 1
PP2A: protein phosphatase 2 A
PR: proline-rich region
PSD-95: postsynaptic density-95
qRT-PCR: quantitative real-time polymerase chain reaction
RNAi: RNA interference
S6K: S6 kinase
S6K C.A.: constitutively active mutant of S6K
S6K K.D.: kinase-dead (inactive) mutant of S6K
Ser127: serine-127
shRNA: short-hairpin RNA
STOP-Tom: STOP-tdTomato
TDL: total dendrite length
TEAD: TEA domain
TGF-β: transforming growth factor-β
Thr389: threonine-389
VGAT: vesicular γ-aminobutyric acid transporter
VGLUT1: vesicular glutamate transporter 1
Yap1: Yes-associated protein 1
