# Supplementary figures and images for: Amot and Yap1 regulate neuronal dendritic tree complexity and locomotor coordination in mice
Source: PLoS Biol. 2019 May 1;17(5):e3000253. doi: 10.1371/journal.pbio.3000253 (PMC6513106; doi:10.1371/journal.pbio.3000253)

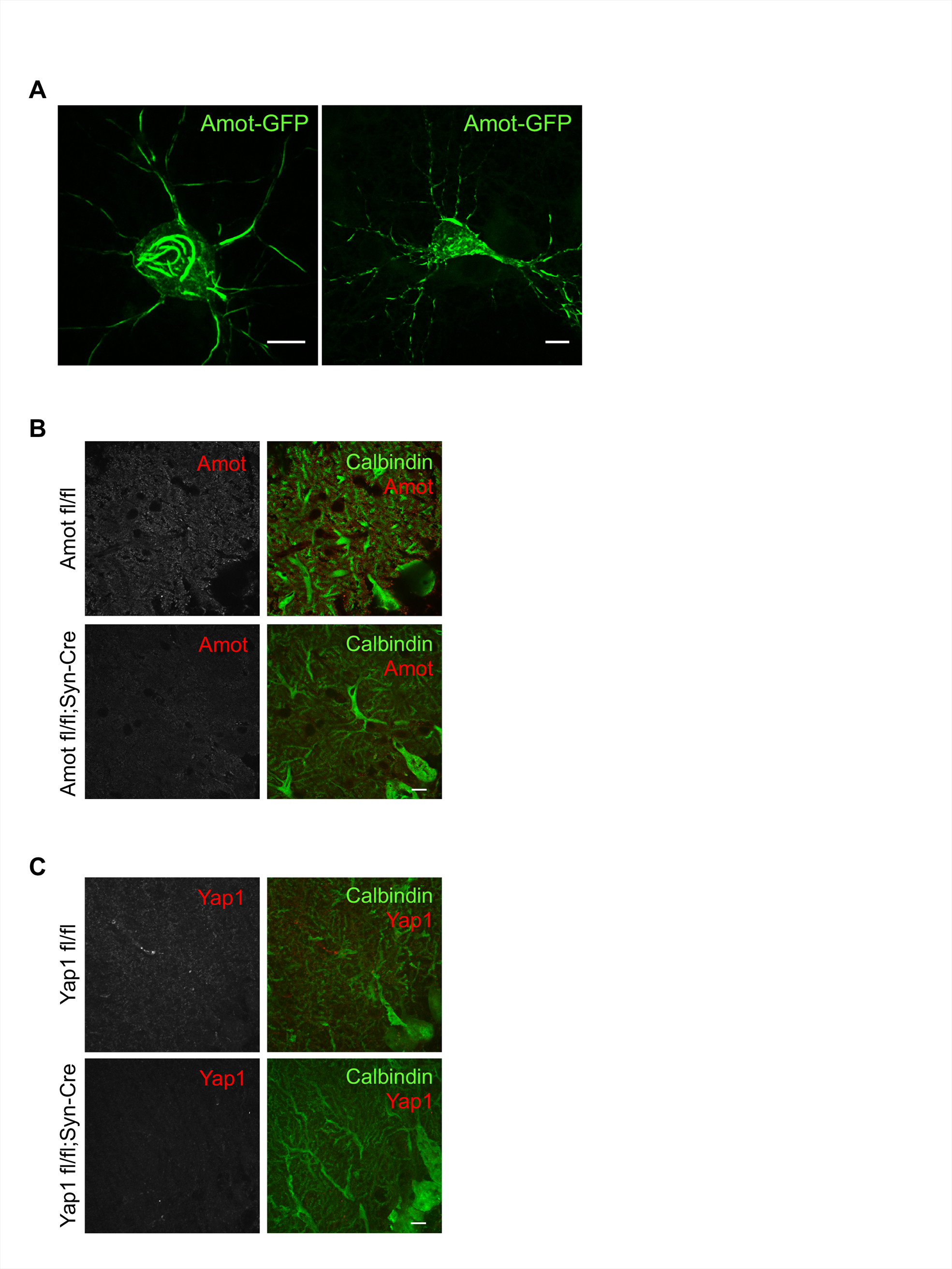

Supplement: S1 Fig — (A) Rat DIV10 hippocampal neurons that expressed Amot–GFP. (B) Cerebellar cross-sections of control Amot fl/fl and Amot fl/fl;Syn-Cre mice stained for Amot (red; the same image acquisition settings) and calbindin (green). (C) Cerebellar cross-sections of control Yap1 fl/fl and Yap1 fl/fl;Syn-Cre mice stained for Yap1 (red; the same image acquisition settings) and calbindin (green). Scale bar = 10 μm in A and 20 μm in B and C. Amot, angiomotin; DIV, day in vitro; GFP, green fluorescent protein; Yap1, Yes-associated protein 1. (TIF) [file pbio.3000253.s001.tif]

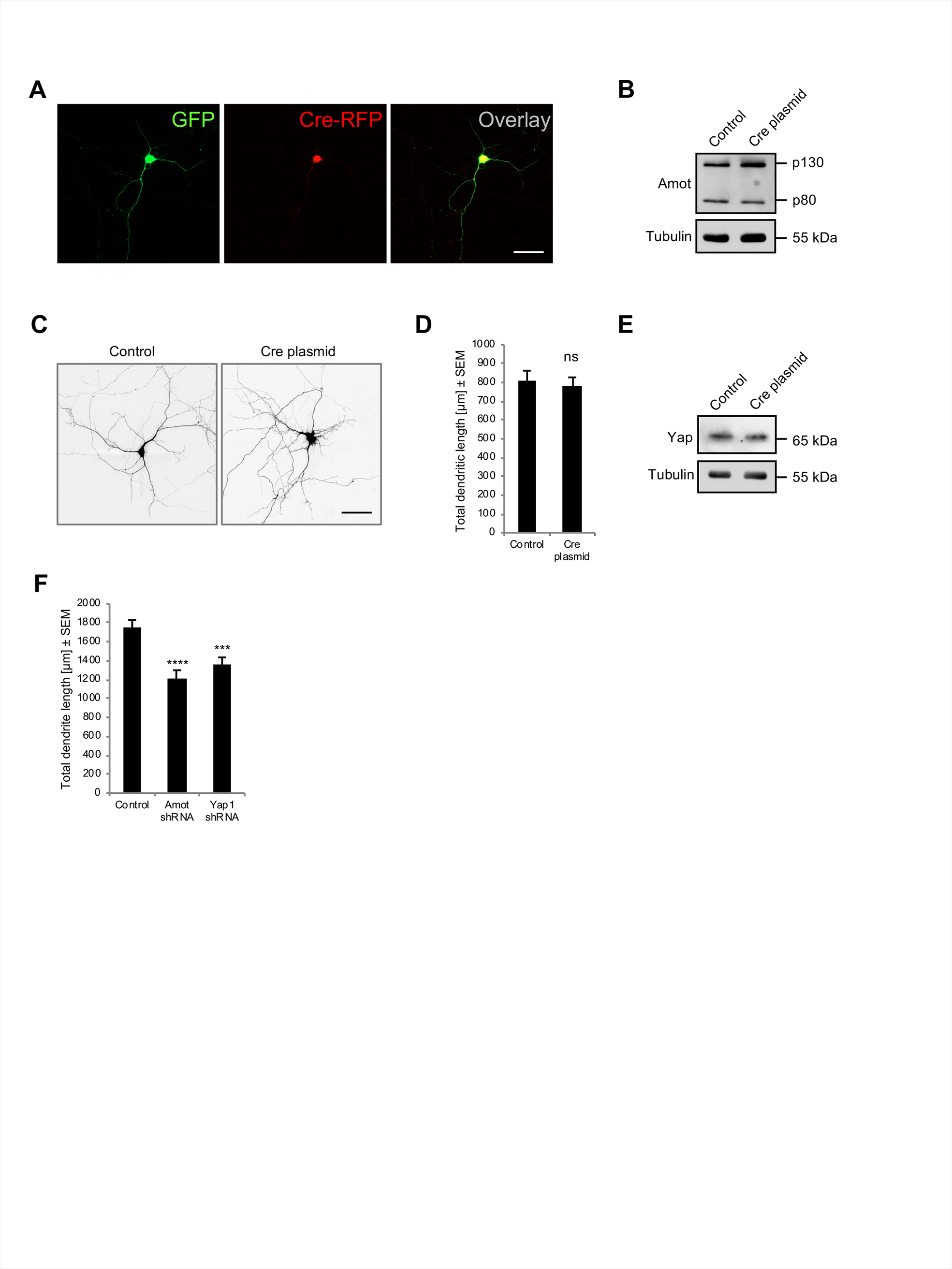

Supplement: S2 Fig — (A) Expression and nuclear localization of Cre in cultured neurons. Representative images of Amot fl/fl mouse hippocampal neurons that were cotransfected with a plasmid that expressed Cre–RFP and a vector with GFP that was used to visualize neuronal morphology. (B) Western blot analysis of Amot expression levels in wild-type mouse cortical neurons that were nucleofected with a control or Cre-expressing plasmid. (C) Representative images of cultured wild-type mouse hippocampal neurons that were transfected with a plasmid that encoded Cre recombinase or a control vector. Scale bars = 100 μm. (D) Quantification of TDL of wild-type mouse hippocampal neurons that were transfected with plasmids that encoded Cre recombinase (n = 46) or a control vector (n = 39). The values are shown as percentage of Control. p = 0.7519. The cells were additionally transfected with a GFP vector to visualize neuronal morphology. Quantification was performed for samples that were obtained from at least three independent cultures. (E) Western blot analysis of Yap1 expression levels in wild-type mouse cortical neurons that were nucleofected with a control or Cre-expressing plasmid. (F) Quantification of TDL of mature rat hippocampal neurons that were depleted of Amot and Yap1. The cells were additionally transfected with a GFP vector to visualize neuronal morphology. The cells were transfected with the indicated plasmids on DIV14 and fixed 4 d later. Control: n = 69; Amot shRNA: n = 60; Yap1 shRNA: n = 37. To Control p < 0.0001, p = 0.0005. Quantification was performed on samples that were obtained from at least three independent cultures. Scale bars = 50 μm. Numerical values that underlie the graph are shown in S1 Data. Statistical significance was analyzed using two-tailed unpaired t tests (D) and one-way analysis of variance followed by Tukey’s post hoc test (F). ***p < 0.001, ****p < 0.0001. Bars represent the mean ± SEM. Amot, angiomotin; DIV, day in vitro; GFP, green fluorescent protein; [file pbio.3000253.s002.tif]

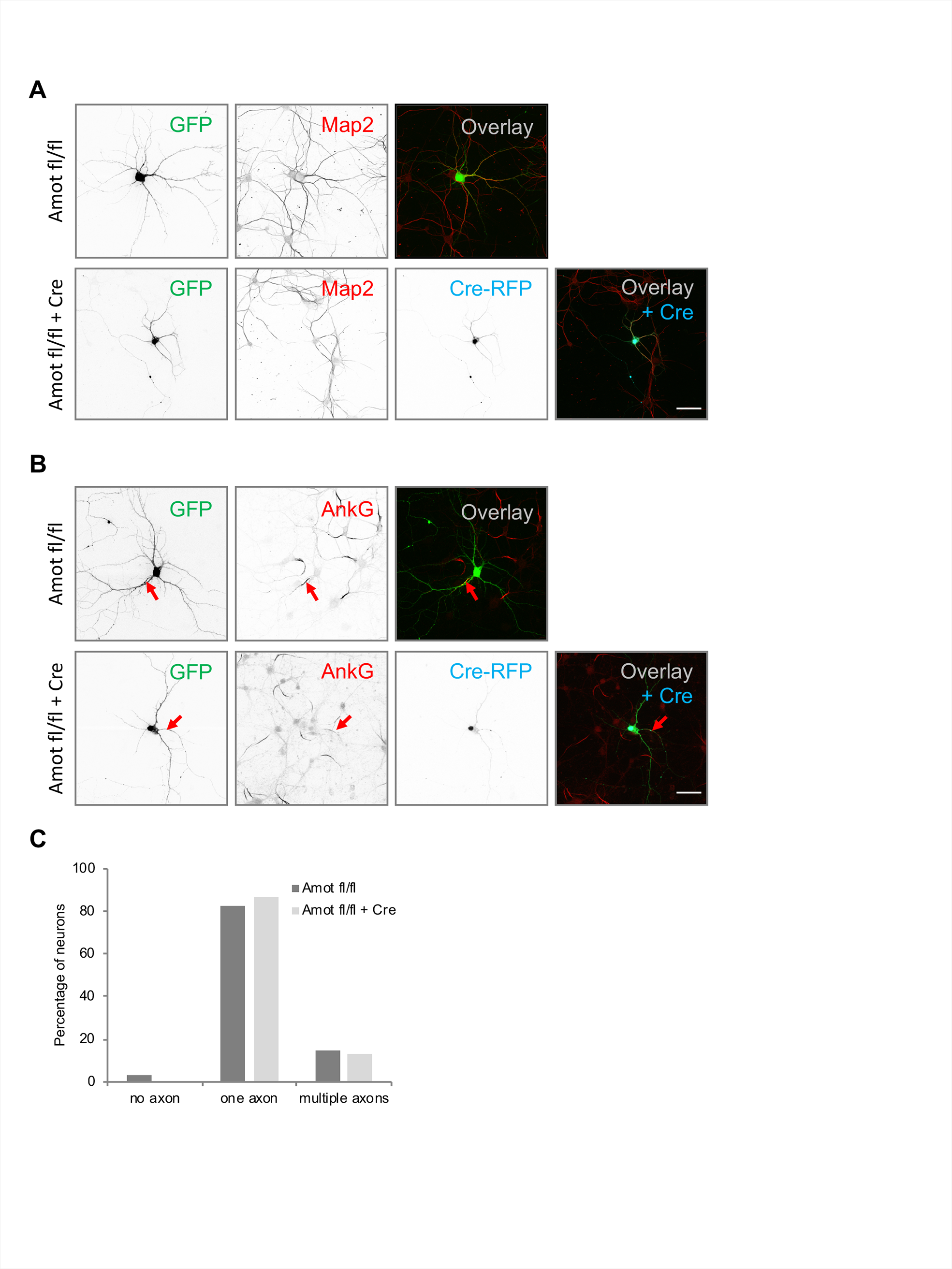

Supplement: S3 Fig — (A, B) Representative images of Amot fl/fl mouse hippocampal neurons that were cotransfected with a plasmid that expressed Cre–RFP or a control vector that were immunolabeled for Map2 (A) or ankyrin G (B). (C) Amot fl/fl hippocampal neurons that were cotransfected with a plasmid that expressed Cre–RFP (n = 33) or a control vector (n = 40), classified according to the number of axons: no axon, single axon, or multiple axons. The cells were cotransfected with a vector that expressed GFP to visualize neuronal morphology. Quantification was performed from at least three independent cultures. Numerical values that underlie the graph are shown in S1 Data. Scale bars = 50 μm. Amot, angiomotin; GFP, green fluorescent protein; Map2, microtubule-associated protein 2; RFP, red fluorescent protein. (TIF) [file pbio.3000253.s003.tif]

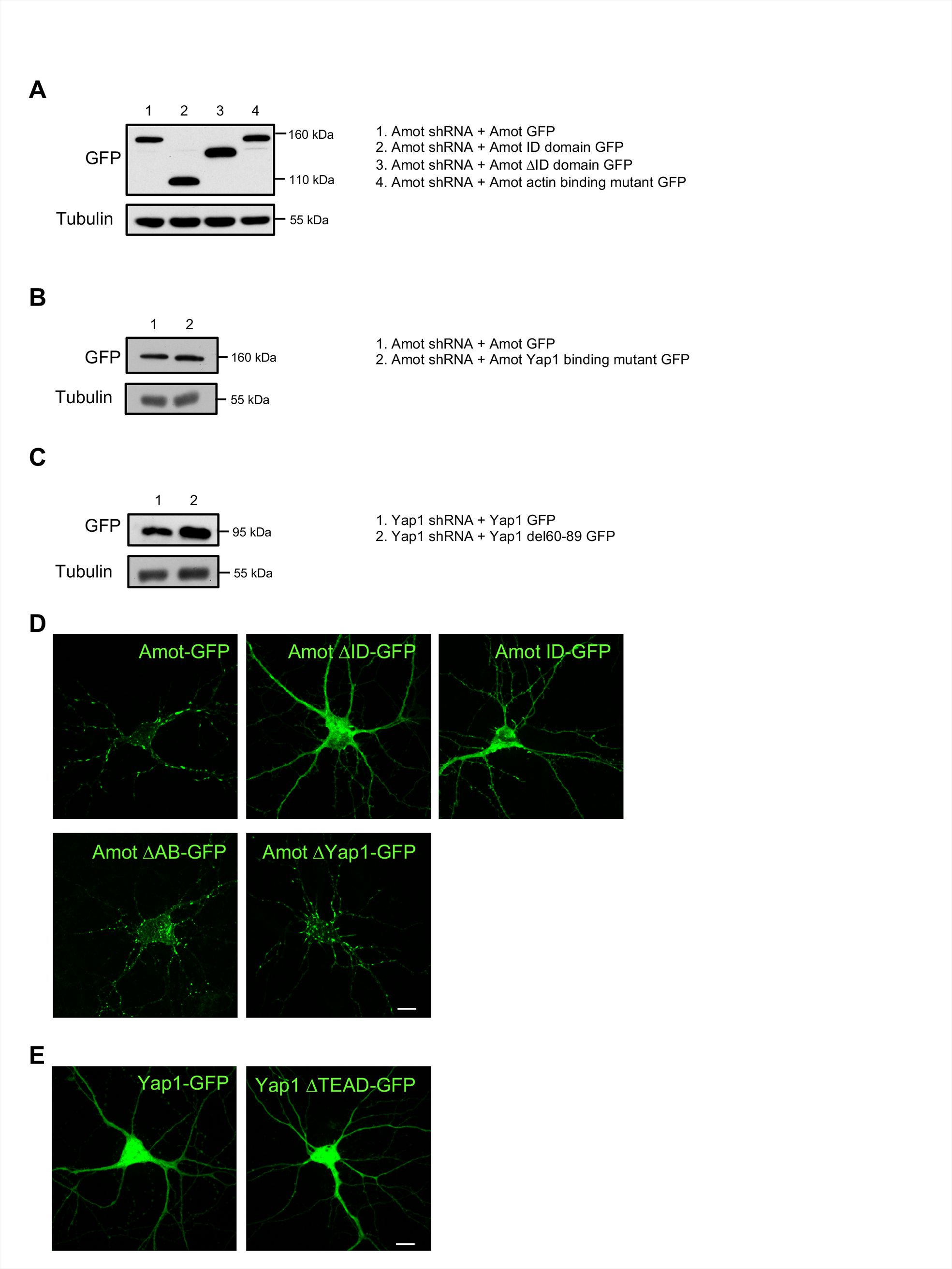

Supplement: S4 Fig — (A-C) Extracts from rat neurons that were cotransfected with plasmids that expressed the indicated constructs were analyzed by western blot using anti-GFP antibody. (D, E) Rat DIV10 hippocampal neurons that expressed the indicated Amot and Yap1 constructs. Scale bars = 10 μm. See Results section for further details. Amot, angiomotin; DIV, day in vitro; GFP, green fluorescent protein; Yap1, Yes-associated protein 1. (TIF) [file pbio.3000253.s004.tif]

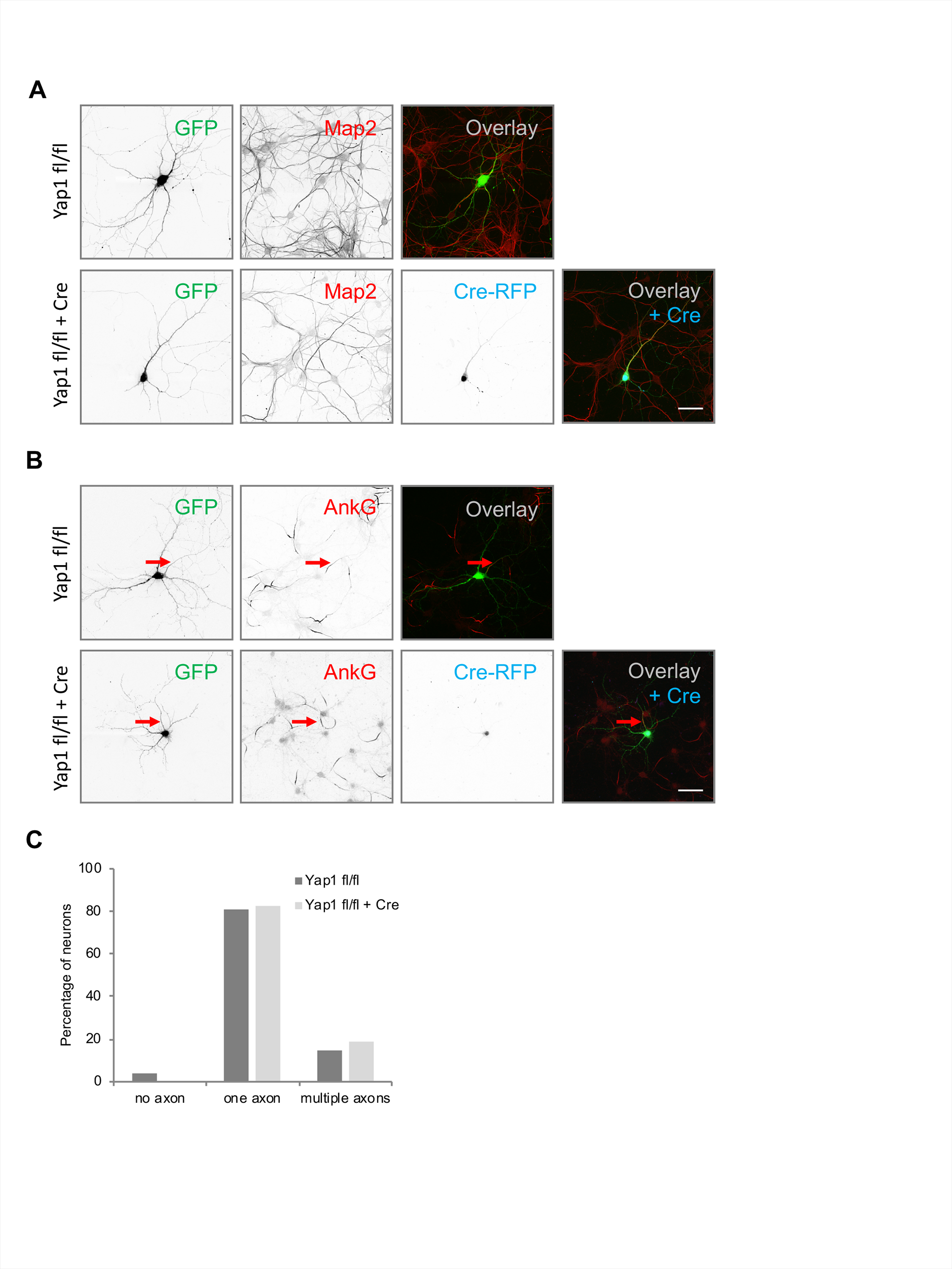

Supplement: S5 Fig — (A, B) Representative images of Yap1 fl/fl mouse hippocampal neurons that were cotransfected with a plasmid that expressed Cre–RFP or a control vector and immunolabeled for Map2 (A) or ankyrin G (B). (C) Yap1 fl/fl hippocampal neurons that were cotransfected with a plasmid that expressed Cre–RFP (n = 53) or a control vector (n = 53), classified according to the number of axons: no axon, single axon, or multiple axons. The cells were cotransfected with a vector that expressed GFP to visualize neuronal morphology. Quantification was performed from at least three independent cultures. Numerical values that underlie the graph are shown in S1 Data. Scale bars = 50 μm. GFP, green fluorescent protein; Map2, microtubule-associated protein 2; RFP, red fluorescent protein; Yap1, Yes-associated protein 1. (TIF) [file pbio.3000253.s005.tif]

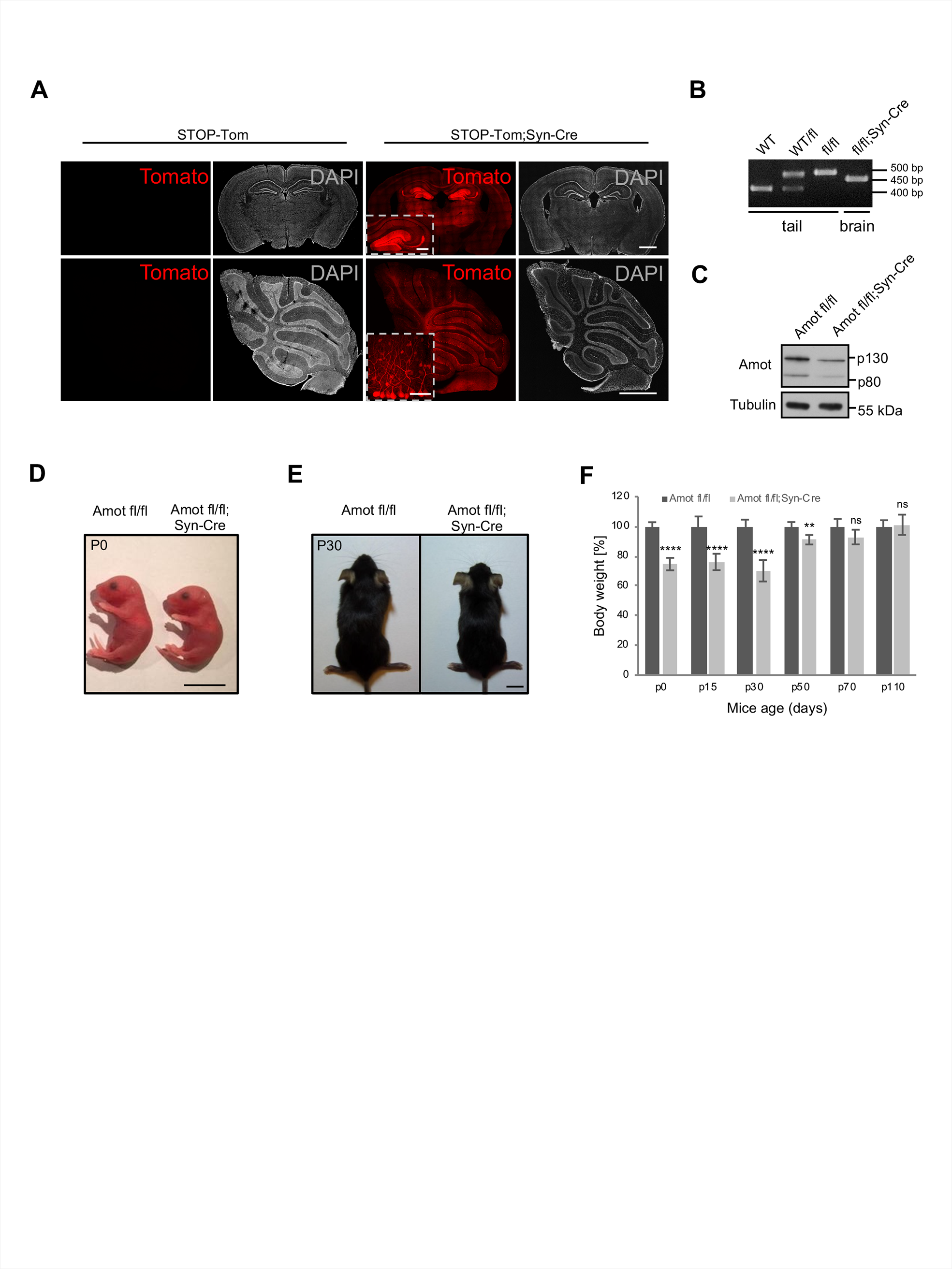

Supplement: S6 Fig — (A) Cross-sections of brains from STOP-Tom;Syn-Cre P30 mice showed high Cre activity in the cerebellum (lower panel) and hippocampus (upper panel). DAPI (gray) was used to visualize brain structures. Scale bars = 1 mm. The insets show higher-magnification images of the hippocampus (upper panel, scale bar = 500 μm) and Purkinje cells (lower panel, scale bar = 50 μm). (B) Confirmation of Amot exon2 excision in the brain of Amot fl/fl;Syn-Cre mice, assessed by PCR analysis of genomic DNA that was obtained from the tail or brain. (C) Reduction of Amot expression in the hippocampus of Amot fl/fl;Syn-Cre P30 mice, analyzed by western blot. (D, E) Images of Amot fl/fl;Syn-Cre and Amot fl/fl control littermates at the neonatal stage (D) or on P30 (E). Scale bars = 1 cm in C and D. (F) Weight analysis of Amot fl/fl and Amot fl/fl;Syn-Cre mice (n = 8, 9, 7, 3, 4, and 4, and n = 6, 4, 9, 6, 6, and 7, respectively) on the indicated days of development. The values are shown as a percentage of control Amot fl/fl mouse weights at the corresponding age. p < 0.0001, p < 0.0001, p < 0.0001, p = 0.0048, p = 0.0561, and p = 0.7799. Numerical values that underlie the graphs are shown in S1 Data. Statistical significance was analyzed using two-tailed unpaired t test. **p < 0.01, ****p < 0.0001. Bars represent the mean ± standard deviation (SD). Amot, angiomotin; fl/fl, Amot homozygote mice; ns, not significant; P, postnatal day; SD, standard deviation; WT, wild-type control; WT/fl, Amot heterozygote mice. (TIF) [file pbio.3000253.s006.tif]

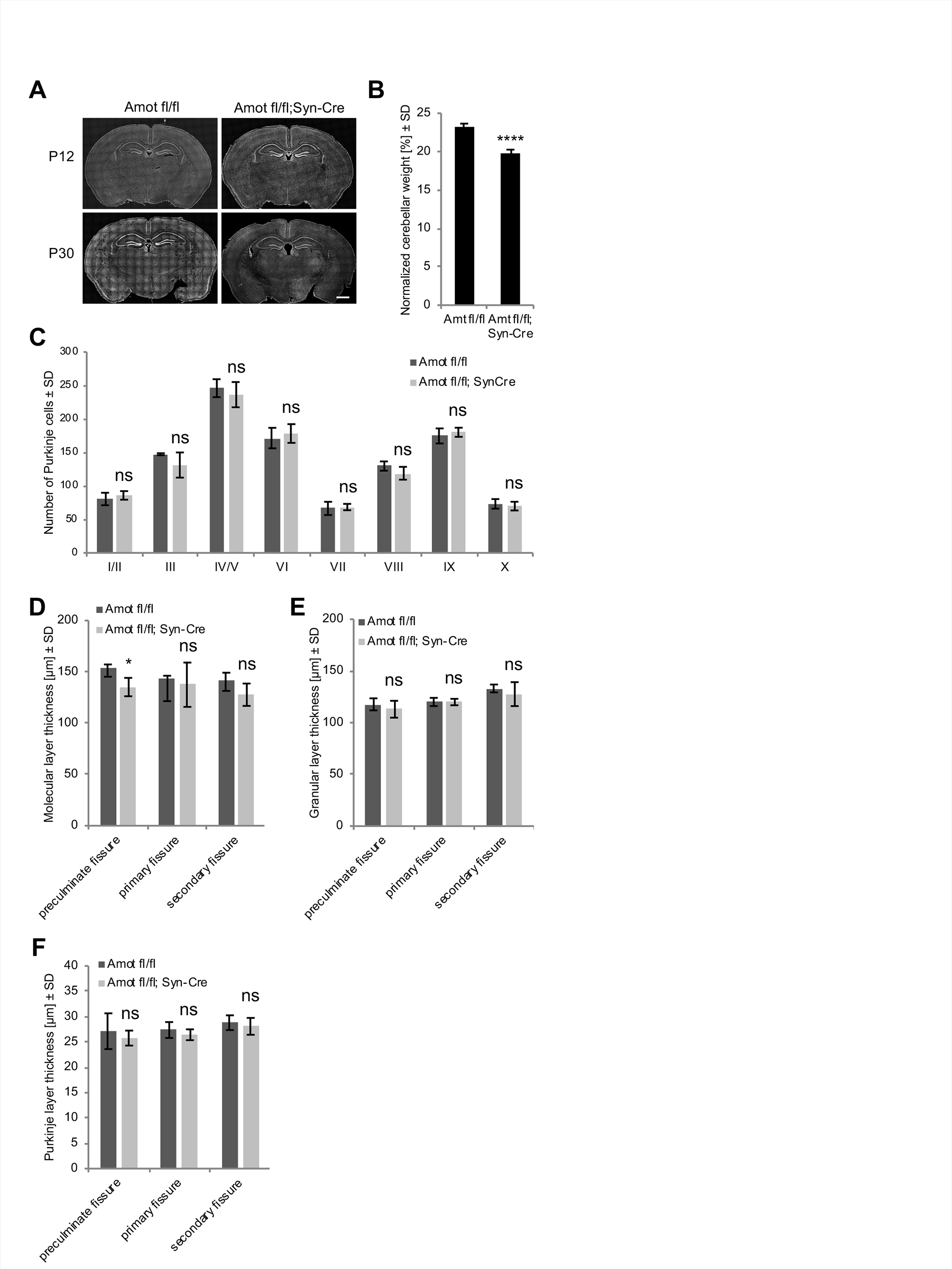

Supplement: S7 Fig — (A) Coronal sections of Amot fl/fl and Amot fl/fl;Syn-Cre brains on P12 and P30 that were stained with DAPI. Scale bars = 1 mm. (B) Quantitative analysis of cerebellar weight of Amot fl/fl (n = 7) and Amot fl/fl;Syn-Cre (n = 3) P150 mice normalized to the whole-brain weight. p < 0.0001. (C) Quantification of Purkinje cells in individual lobes of the cerebellum of Amot fl/fl (n = 3 mice and at least three sections per mouse) and Amot fl/fl;Syn-Cre (n = 5 mice and at least three sections per mouse) P30 mice. Numbers indicate the lobes. p = 0.3669, p = 0.2006, p = 0.4707, p = 0.5413, p = 0.6975, p = 0.1447, p = 0.4154, p = 0.5165. (D-F) Thickness of the molecular (D), granular (E), and Purkinje (F) cell layers in the cerebellum of Amot fl/fl (n = 3 mice and at least three sections per mouse) and Amot fl/fl;Syn-Cre (n = 5 mice and at least three sections per mouse) P30 mice measured at the preculminate, primary, and secondary fissures. p = 0.0135, p = 0.6818, and p = 0.0859 in D; p = 0.4169, p = 0.9693, and p = 0.4702 in E; p = 0.4615, p = 0.3473, and p = 0.5603 in F. Numerical values that underlie the graphs are shown in S1 Data. Statistical significance was analyzed using two-tailed unpaired t test. *p < 0.05, ****p < 0.0001. Bars represent the mean ± SD. ns, not significant; P, postnatal day; SD, standard deviation. (TIF) [file pbio.3000253.s007.tif]

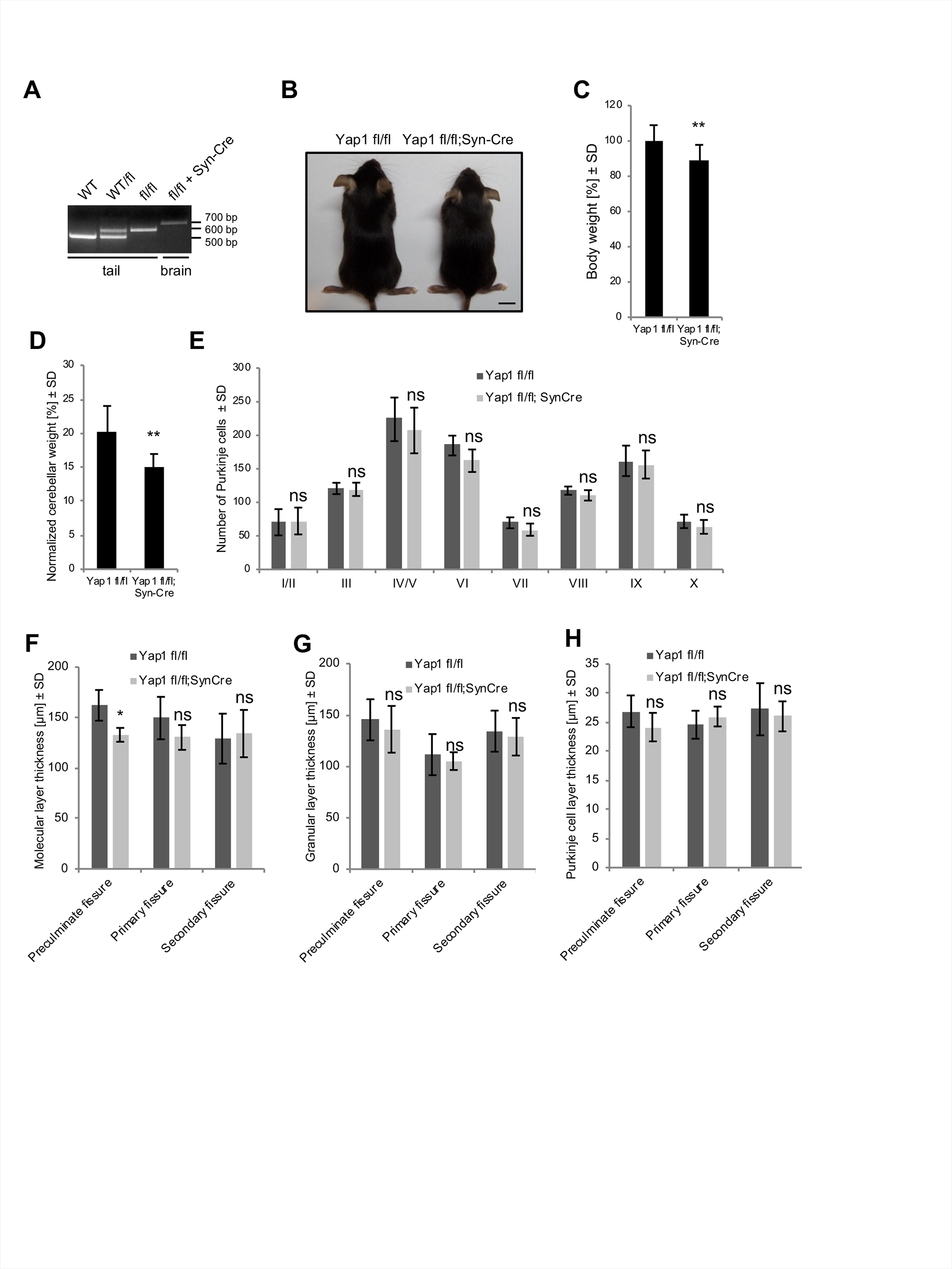

Supplement: S8 Fig — (A) Confirmation of Yap1 exon1–2 excision in the brain of Yap1 fl/fl;Syn-Cre mice, assessed by PCR analysis of genomic DNA that were obtained from the tail or brain. (B) Yap1 fl/fl;Syn-Cre P30 mice appeared to be smaller than Yap1 fl/fl control littermates. (C) Weight analysis of Yap1 fl/fl (n = 20) and Yap1 fl/fl;Syn-Cre (n = 9) P30 mice. p = 0.0036. (D) Quantitative analysis of cerebellar weight of Yap1 fl/fl (n = 7) and Yap1 fl/fl;Syn-Cre (n = 9) P150 mice normalized to the whole-brain weight. p = 0.0023. (E) Quantification of Purkinje cells in individual lobes of Yap1 fl/fl (n = 4 mice and at least three sections per mouse) and Yap1 fl/fl;Syn-Cre (n = 4 mice and at least three sections per mouse) P30 mice. Numbers indicate the lobes. p = 0.9206, p = 0.6583, p = 0.4626, p = 0.0574, p = 0.1038, p = 0.1003, p = 0.8263, p = 0.2776. (F-H) Thickness of the molecular (F), granular (G) and Purkinje (H) cell layers in the cerebellum of Yap1 fl/fl (n = 4 mice and at least three sections per mouse) and Yap1 fl/fl;Syn-Cre (n = 4 mice and at least three sections per mouse) P30 mice measured at the preculminate, primary, and secondary fissures. p = 0.0128, p = 0.2086, and p = 0.7616 in F; p = 0.4679, p = 0.5387, and p = 0.6736 in G; p = 0.1885, p = 0.3746, and p = 0.6526 in H. Numerical values that underlie the graphs are shown in S1 Data. Statistical significance was analyzed using two-tailed unpaired t test. *p < 0.05, **p < 0.01. Bars represent the mean ± SD. fl/fl, Yap1 homozygote mice; ns, not significant; P, postnatal day; SD, standard deviation; WT, wild-type control; WT/fl, Yap1 heterozygote mice; Yap1, Yes-associated protein 1. (TIF) [file pbio.3000253.s008.tif]

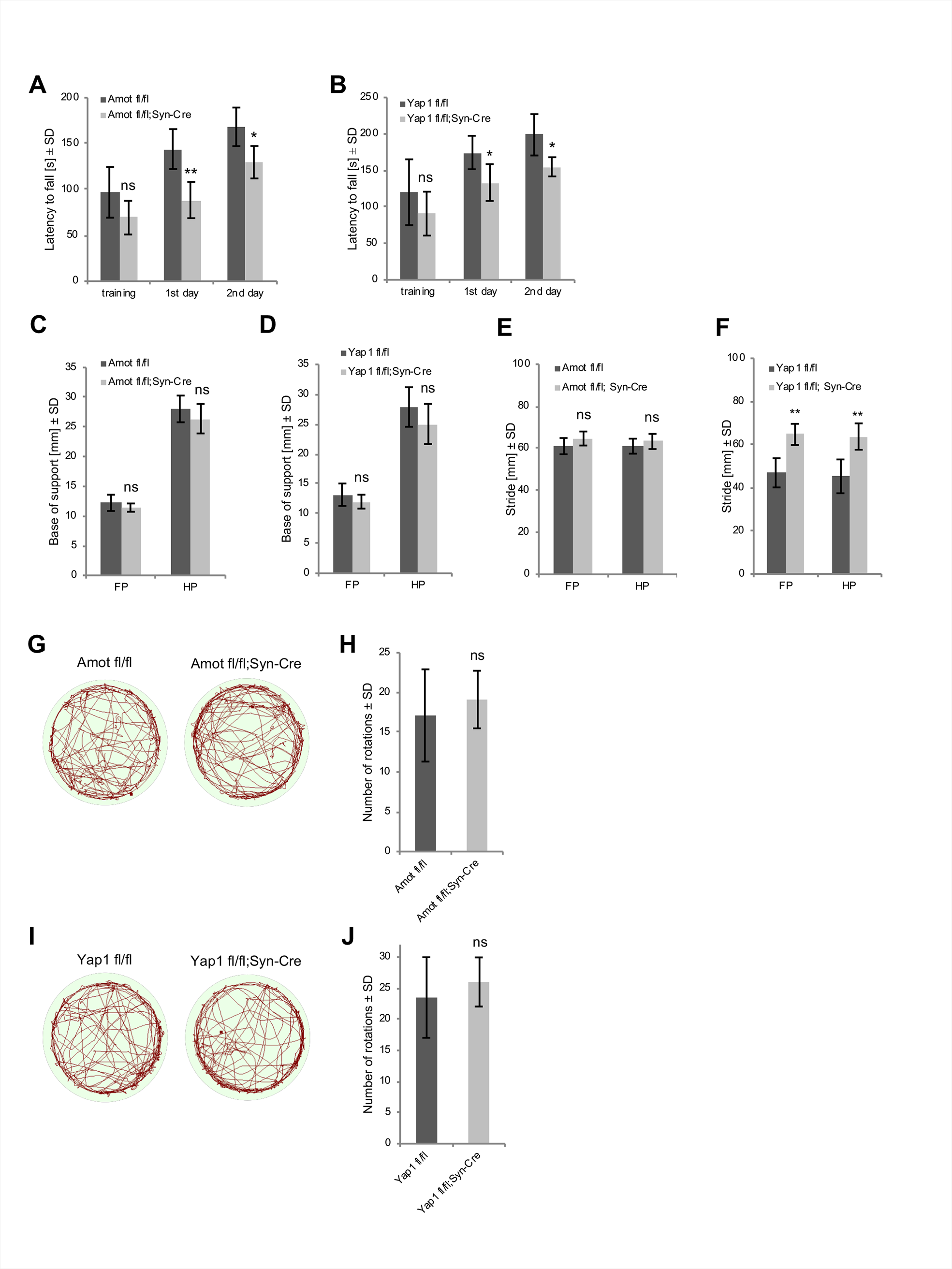

Supplement: S9 Fig — (A) Locomotor coordination in Amot fl/fl (n = 5) and Amot fl/fl;Syn-Cre (n = 5) P100 mice, reflected by the latency to fall from the rotarod. p = 0.0988, p = 0.0029, and p = 0.0137. (B) Locomotor coordination in Yap1 fl/fl (n = 5) and Yap1 fl/fl;Syn-Cre (n = 5) P80 mice, reflected by the latency to fall from the rotarod. p = 0.2611, p = 0.0270, and p = 0.0138. (C) CatWalk gait analysis of BOS in Amot mutant mice (Amot fl/fl: n = 11; Amot fl/fl;Syn-Cre: n = 5; p = 0.2418 and p = 0.2081) and (D) CatWalk gait analysis of BOS in Yap1 mutant mice (Yap1 fl/fl: n = 4; Yap1 fl/fl;Syn-Cre, n = 6; p = 0.2571 and p = 0.2243). (E) CatWalk gait analysis of stride length in Amot mutant mice (Amot fl/fl: n = 11; Amot fl/fl;Syn-Cre: n = 5; p = 0.0922 and p = 0.2643). (F) CatWalk gait analysis of stride length in Yap1 mutant mice (Yap1 fl/fl: n = 4; Yap1 fl/fl;Syn-Cre: n = 6; p = 0.0013 and p = 0.0030). (G-J) Open-field analysis of Amot (G, H) and Yap1 (I, J) mutant mice (Amot fl/fl;Syn-Cre: n = 9; Amot fl/fl: n = 9; Yap1 fl/fl;Syn-Cre: n = 6; Yap1 fl/fl: n = 8). p = 0.3930 in H and p = 0.4220 in J. Numerical values that underlie the graphs are shown in S1 Data. Statistical significance was analyzed using two-tailed unpaired t tests. *p < 0.05, **p < 0.01. Bars represent the mean ± SD. Amot, angiomotin; BOS, base of support; FP, forepaws; HP, hind paws; ns, not significant; P, postnatal day; SD, standard deviation; Yap1, Yes-associated protein 1. (TIF) [file pbio.3000253.s009.tif]

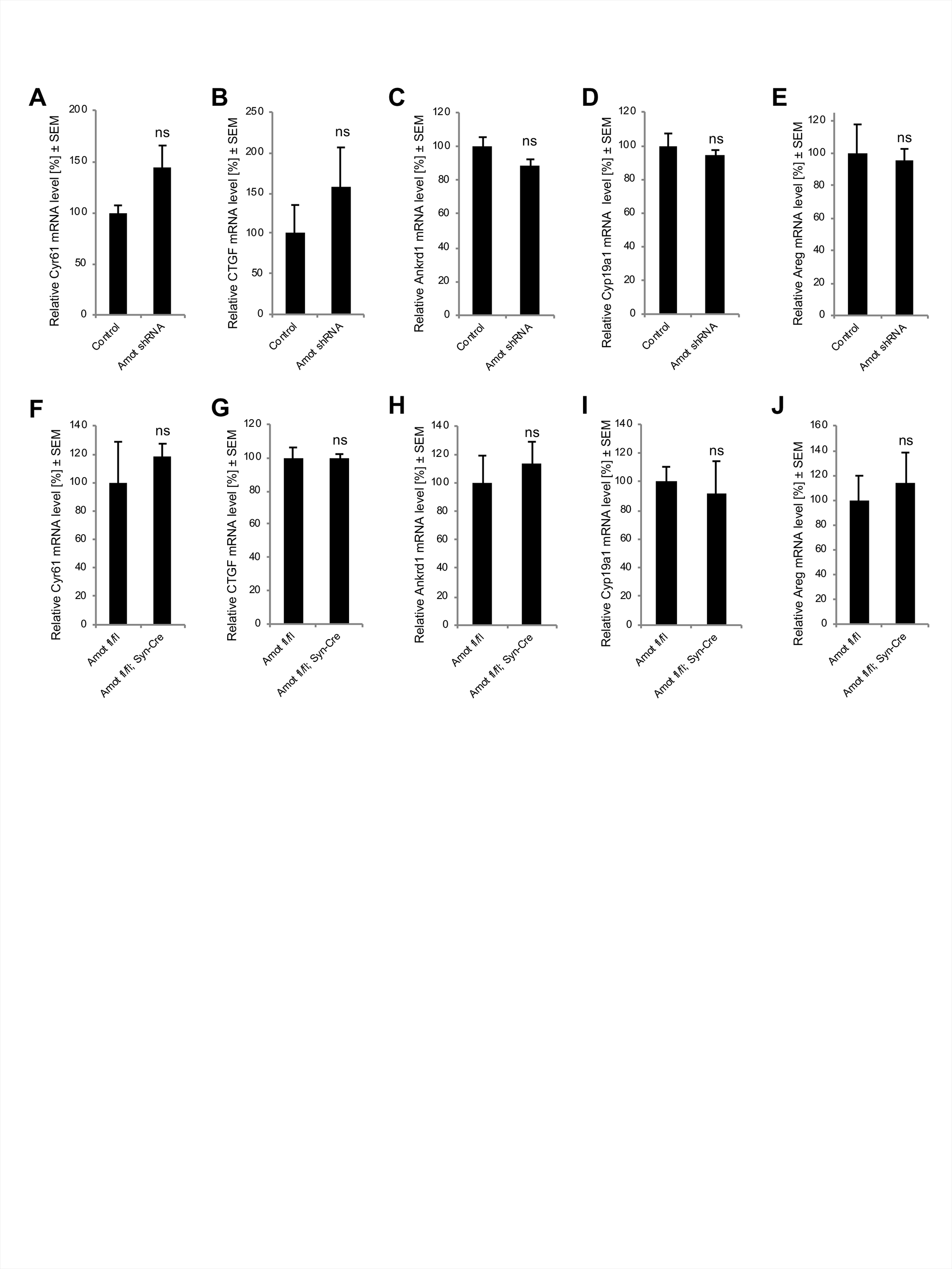

Supplement: S10 Fig — (A-J) qRT-PCR analysis of Cyr61 (A, F), CTGF (B, G), Ankrd1 (C, H), Cyp19a1 (D, I), and Areg (E, J) expression in rat cortical neurons (A-E) with Amot knockdown (n = 3/group) and cerebellar homogenates (F-J) of Amot knockouts (n = 6 in F and G; n = 4 in H, I and J) or control P30 mice (n = 4 in F and G; n = 6 in H, I and J). p = 0.0599, p = 0.3478, p = 0.1014, p = 0.4786, p = 0.8153, p = 0.057, p = 0.9804, p = 0.6133, p = 0.7081, p = 0.6600. Numerical values that underlie the graphs are shown in S1 Data. Statistical significance was analyzed using two-tailed unpaired t tests. Bars represent the mean ± SEM. Amot, angiomotin; Ankrd1, ankyrin repeat domain 1; Areg, amphiregulin; CTGF, connective tissue growth factor; Cyp19a1, cytochrome P450 family 19 subfamily A member 1; Cyr61, cysteine-rich 61; ns, not significant; P, postnatal day; qRT-PCR, quantitative real-time polymerase chain reaction; SEM, standard error of the mean. (TIF) [file pbio.3000253.s010.tif]

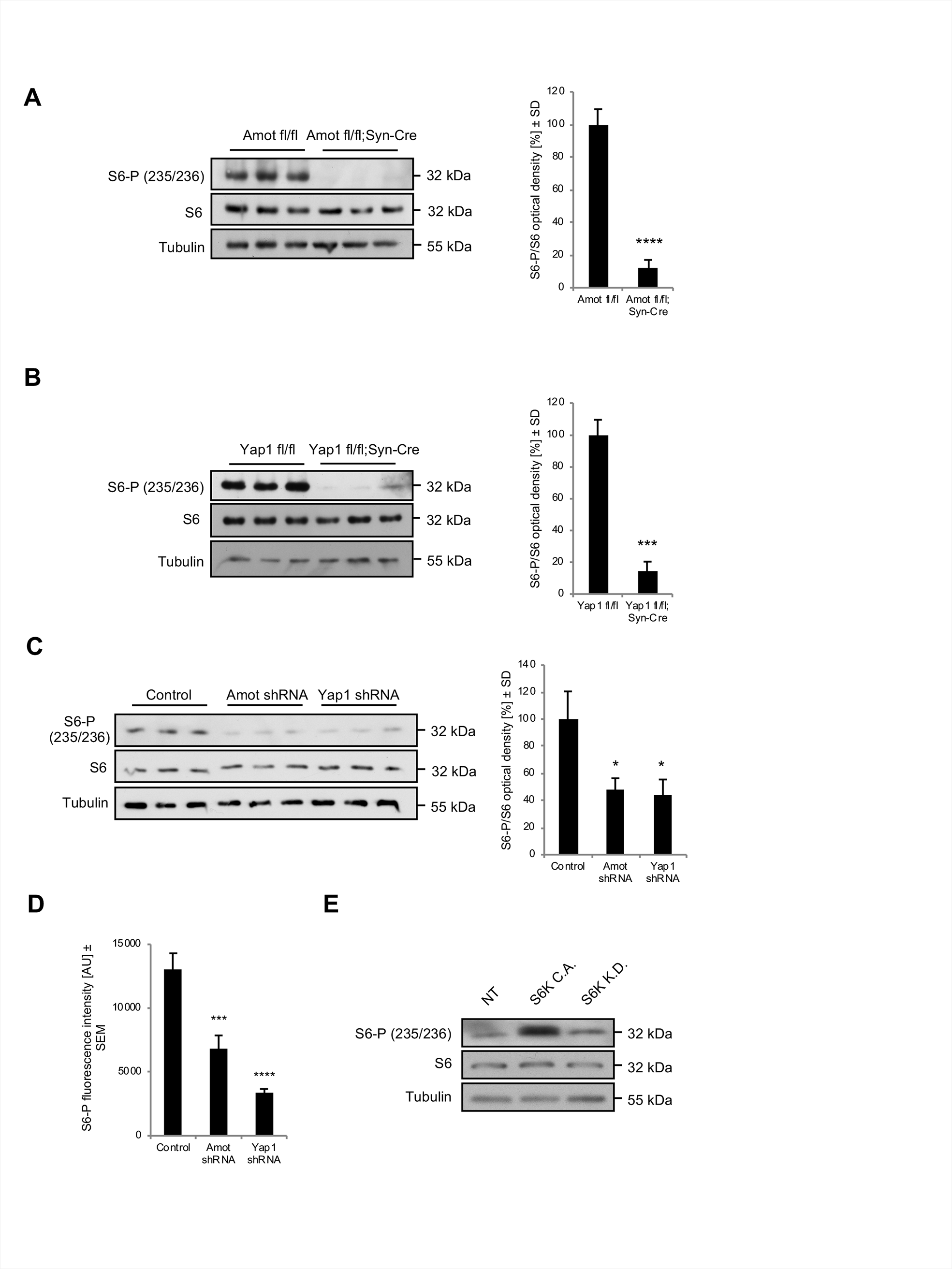

Supplement: S11 Fig — (A-C) Western blot analysis and quantification of S6 phosphorylation at Ser235/236 in cerebellum homogenates of Amot fl/fl and Amot fl/fl;Syn-Cre mice (A) and Yap1 fl/fl and Yap1 fl/fl;Syn-Cre mice (B) and in rat cortical neurons that were nucleofected with Amot shRNA, Yap1 shRNA, or control plasmid (C). p < 0.0001, p = 0.0002, p = 0.0155, p = 0.0147. Tubulin is shown as a loading control. The signal was normalized to the control. (D) Quantification of phosphorylated S6 (Ser235/236) fluorescence intensity in cultured hippocampal neurons that were transfected with control plasmid (n = 63), Amot shRNA (n = 42), and Yap1 shRNA (n = 41). p = 0.0005 and p < 0.0001. (E) Ectopic expression of S6K C.A. but not S6K K.D. led to an increase in S6 phosphorylation at Ser235/236 in HEK-293 cells. Numerical values that underlie the graphs are shown in S1 Data. Statistical significance was analyzed using two-tailed unpaired t tests. *p < 0.05, ***p < 0.001, and ****p < 0.001. Bars represent the mean ± SD. Amot, angiomotin; HEK-293, human embryonic kidney 293; NT, not transfected; S6K C.A., constitutively active S6 kinase; S6K K.D., kinase-dead (inactive) mutant S6 kinase; SD, standard deviation; Ser, serine; shRNA, short-hairpin RNA; Yap1, Yes-associated protein 1. (TIF) [file pbio.3000253.s011.tif]
